# TLR7 Controls VSV Replication in CD169^+^ SCS Macrophages and Associated Viral Neuroinvasion

**DOI:** 10.3389/fimmu.2019.00466

**Published:** 2019-03-15

**Authors:** Gülhas Solmaz, Franz Puttur, Marcela Francozo, Marc Lindenberg, Melanie Guderian, Maxine Swallow, Vikas Duhan, Vishal Khairnar, Ulrich Kalinke, Burkhard Ludewig, Björn E. Clausen, Hermann Wagner, Karl S. Lang, Tim D. Sparwasser

**Affiliations:** ^1^Institute of Infection Immunology, TWINCORE, Centre for Experimental and Clinical Infection Research, A Joint Venture Between the Medical School Hannover and the Helmholtz Centre for Infection Research (HZI), Hannover, Germany; ^2^Institute of Immunology of the University Hospital in Essen, Medical Faculty, University of Duisburg-Essen, Essen, Germany; ^3^Institute of Experimental Infection Research, TWINCORE, Centre for Experimental and Clinical Infection Research, A Joint Venture Between the Hannover Medical School and the Helmholtz Centre for Infection Research (HZI), Hannover, Germany; ^4^Institute of Immunobiology, Kantonsspital St. Gallen, St. Gallen, Switzerland; ^5^Institute for Molecular Medicine, University Medical Center of the Johannes Gutenberg-University Mainz, Mainz, Germany; ^6^Institute for Medical Microbiology, Immunology and Hygiene, Technical University Munich, Munich, Germany; ^7^Department of Medical Microbiology and Hygiene, University Medical Center of the Johannes Gutenberg University Mainz, Mainz, Germany

**Keywords:** pattern recognition receptors, Toll-like receptor 7, vesicular stomatitis virus, virus replication, innate immunity, subcapsular sinus macrophages, subcutaneous infection, conditional knock-out mice

## Abstract

Vesicular stomatitis virus (VSV) is an insect-transmitted rhabdovirus that is neurovirulent in mice. Upon peripheral VSV infection, CD169^+^ subcapsular sinus (SCS) macrophages capture VSV in the lymph, support viral replication, and prevent CNS neuroinvasion. To date, the precise mechanisms controlling VSV infection in SCS macrophages remain incompletely understood. Here, we show that Toll-like receptor-7 (TLR7), the main sensing receptor for VSV, is central in controlling lymph-borne VSV infection. Following VSV skin infection, TLR7^−/−^ mice display significantly less VSV titers in the draining lymph nodes (dLN) and viral replication is attenuated in SCS macrophages. In contrast to effects of TLR7 in impeding VSV replication in the dLN, TLR7^−/−^ mice present elevated viral load in the brain and spinal cord highlighting their susceptibility to VSV neuroinvasion. By generating novel TLR7 floxed mice, we interrogate the impact of cell-specific TLR7 function in anti-viral immunity after VSV skin infection. Our data suggests that TLR7 signaling in SCS macrophages supports VSV replication in these cells, increasing LN infection and may account for the delayed onset of VSV-induced neurovirulence observed in TLR7^−/−^ mice. Overall, we identify TLR7 as a novel and essential host factor that critically controls anti-viral immunity to VSV. Furthermore, the novel mouse model generated in our study will be of valuable importance to shed light on cell-intrinsic TLR7 biology in future studies.

## Introduction

Vesicular stomatitis virus (VSV) is a prototypic member of the Rhabdovirus family that consists of neurotropic viruses including Rabies virus. VSV can be transmitted horizontally from insects to mammals (1, 2), infecting host cells to shut down protein synthesis and facilitate the production of new virus particles (3). Even though primary VSV infection commences in peripheral tissues, breaches in immune barrier integrity impair viral control, enabling the virus to spread to peripheral nerves and subsequently invade the central nervous system (CNS) (4). The neurotropism exhibited by VSV causes paralytic symptoms in mammals, including mice. Therefore, it serves as an ideal model for investigating immunity to human neurotropic viruses like rabies virus (5). In mice, immunity to VSV involves a robust, multifaceted cytokine, and cellular response restricting viral replication after infection (6). Immune protection and host survival are strongly determined by the induction of an efficient type-I interferon (IFN) response (7, 8). This response is strictly regulated by viral sensing pattern recognition receptors (PRRs), mainly endosomal Toll-like receptor-7 (TLR7), and cytosolic retinoic acid–inducible gene I (RIG-I) receptors (9, 10). Dendritic cells (DC), in particular plasmacytoid DC (pDC), utilize TLR7 to sense VSV, and are major producers of early type-I IFNs following systemic VSV infection (9, 11). Prior to systemic infection, pDC ablation by diphtheria toxin (DT) treatment in BDCA2-DTR transgenic mice enhances early VSV replication in the spleen, while the accumulation and survival of VSV-specific cytotoxic T cells are significantly reduced (12). Simultaneously, adoptive transfer of TLR7-sufficient pDC into TLR/RIG-I–deficient hosts prolongs survival, inducing an antiviral state in peripheral organs, however, eventually the mice succumb to VSV-induced CNS neuroinvasion (13). Hence, pDC curtail VSV replication in peripheral organs by early type-I IFN induction but fail to endow complete protection against VSV dissemination to the CNS.

In addition to pDC, lymph node (LN) subcapsular sinus (SCS) macrophages produce robust type-I IFN upon peripheral VSV infection and simultaneously enable productive VSV replication in immunocompetent wild-type (Wt) mice (14). Clodronate depletion of macrophages prior to subcutaneous (s.c.) VSV infection reduces type-I IFN levels comparable to the combined depletion of macrophages and pDC (14) and triggers CNS disease (14, 15). Thus, SCS macrophage-derived type-I IFN is neuroprotective and central in preventing VSV dissemination to the peripheral nerves (14). Simultaneously, in a systemic VSV infection model, it was demonstrated that splenic CD169^+^ metallophilic macrophages sustain enforced VSV replication, mechanistically controlled by *Usp18* gene expression (16). This facilitates prolonged antigen presentation, induction of protective neutralizing antibody responses and protects mice from CNS disease (16). However, mice succumb to CNS disease even in the presence of high neutralizing antibody titers (14). In line with these findings, it was shown that VSV skin infection of mice retaining B cells but lacking the capacity to produce antibodies induces comparable type-I IFN responses and results in equal survival rate compared to Wt mice (17). Additionally, studies by Mosemann et al. showed that B cells provide a critical source of lymphotoxin α_1_β_2_ (LTα_1_β_2_), required for adequate SCS macrophage differentiation, sustained permissiveness to VSV replication and robust type-I IFN production (17).

TLR7 and its downstream adaptor protein myeloid differentiation primary response gene 88 (MyD88) are critical for type-I IFN production. TLR7^−/−^ and MyD88^−/−^ mice intravenously (i.v.) infected with VSV have dramatically reduced serum IFN levels (9). Intriguingly, how the TLR7/MyD88 pathway orchestrates VSV infection in dLN and subsequent type-I IFN responses after skin infection remains unresolved and warrants an in-depth investigation. Moreover, this route of infection depicts the most natural course of VSV transmission as a prototypic arbovirus. Hence, understanding the functional contribution of TLR7 sensing in skin cells prior to the entry of virus in the dLN would provide novel insights into the cell-specific TLR7 immune function over the entire course of natural VSV infection. Our results demonstrated that mice deficient in TLR7/MyD88 signaling develop hind limb paralysis and succumb to CNS disease after s.c. virus administration via the foot skin. Interestingly, VSV infection was severely hindered in the dLN of TLR7^−/−^ and MyD88^−/−^ mice. Given that TLR7 is broadly expressed on diverse cell types including stromal and lymphoid cells, understanding cell-specific TLR7/MyD88 function in regulating immune protection against VSV is of vital importance. To this aim, we generated novel transgenic TLR7^fl/fl^ mice to interrogate how TLR7 function in different cellular compartments regulates immunity to cutaneous VSV infection. By utilizing conditional knockout mice lacking TLR7 function in CD169^+^ lymphoid macrophages, we demonstrated that deletion of intrinsic TLR7 signaling in SCS macrophages results in a similar but less pronounced reduction in viral titers in dLN. Overall, we establish that TLR7 is an essential host factor controlling the permissiveness of SCS macrophages to productive VSV infection in skin dLN and thereby protecting mice from VSV-induced neurovirulence.

## Materials and Methods

### Mice

All mice used in this study were maintained and bred under specific pathogen free (SPF) conditions in the animal facilities of Twincore (Hannover, Germany) and Helmholtz Center for Infection Research (Braunschweig, Germany). For all experimental groups, age- and sex- matched mice were used (6–14 weeks old). TLR7^−/−^ (18) and MyD88^−/−^ (19) mice were kindly provided by S. Akira. Itgax (CD11c)-Cre mice (20) were provided by B. Reizis, Lysozyme M-Cre mice (21) by I. Förster, CD19-Cre mice (22) by S. Lienenklaus, Podoplanin-Cre mice (23) by B. Ludewig, Langerin-Cre mice (24) and Langerin- DTR:eGFP mice (25) by B.E. Clausen, and RFP flox mice (26) by H.J. Fehling. CD169-Cre mice were provided by the RIKEN BRC through the National Bio-Resource Project of the MEXT, Japan (27). All mice were maintained on the C57BL/6 genetic background.

### Generation of TLR7^fl/fl^ Mice

TLR7^fl/fl^ mice were generated by TaconicArtemis GmbH (Cologne, Germany) as a part of the collaboration with H. Wagner (Technical University of Munich, Munich, Germany). Exon 3 of mouse *TLR7* gene was targeted to generate a conditional allele of *TLR7* by homologous recombination in C57BL/6N embryonic stem (ES) cells. Excision of exon 3 was predicted to cause removal of most of the open reading frame of *TLR7* gene and thereby resulted in loss of function of the gene. The targeting vector consisted of one loxP site and FRT-flanked neomycin resistance cassette inserted upstream of targeted exon as well as a F3-flanked puromycin resistance cassette and one loxP site inserted downstream of exon 3 of *TLR7* allele to increase the efficiency of co-recombination of both loxP sites. The targeting vector also comprised of an ampicillin resistance gene and herpes simplex virus thymidine kinase (Tk) gene driven by phosphoglycerate kinase (pgk) promoter. The targeting vector was linearized with NotI and transfected into C57BL/6N ES cells by electroporation. G418 and ganciclovir resistant ES cells were selected as correctly targeted colonies and screened by Southern blots. TLR7^fl/fl^ mice were backcrossed with C57BL/6J mice for at least 10 generations and were bred as homozygotes.

### Viruses

VSV serotype Indiana (VSV-Indiana; Mudd-Summers-derived clone) (28) was originally obtained from D. Kolakofsky (University of Geneva, Switzerland). Luciferase expressing VSV-Indiana (VSV-Luciferase) (29) was kindly provided by S. Whelan (Harvard Medical School, USA) and VSV-enhanced GFP (VSV-eGFP) (30) by J.K. Rose (Yale University School of Medicine, New Haven, USA). All VSV strains were propagated on BHK-21 cells and infectivity of virus stocks was determined by plaque formation on green monkey kidney Vero (ATCC CCL-81) cells.

### Standard VSV Plaque Assay

VSV titers from infected organs were determined by homogenizing the organs in 0.5 ml MEM medium with a handheld homogenizer, followed by standard plaque assay, as described before (8).

### Subcutaneous VSV Infection via the Foot Skin

Groups of age- and sex- matched mice were anesthetized with vaporized inhaled isoflurane for a short time. Immediately, mice were infected with 10 μl of 5 × 10^5^ pfu of VSV-Indiana (or VSV-Luciferase for *in vivo* imaging analysis) s.c. via the dorsal foot skin of both hind limbs. For some experiments, control (mock treated) mice were treated s.c. with 10 μl of sterile PBS. For some experiments, intradermal infection with VSV-Indiana (5 × 10^5^ pfu in 10 μl of PBS) in each ear pinna was performed after anesthetizing mice with ketamine/xylazin. Unless otherwise stated, mice were sacrificed by CO_2_ asphyxiation at 12 h p.i. and organs were harvested for further assays. Mice in the survival experiments were infected with 5 × 10^6^ pfu of VSV-Indiana and checked daily from day 5 p.i. Mice showing clinical signs of viral neuroinvasion were removed from the experiment and euthanized.

### Generation of Bone Marrow Chimeras

Bone marrow (BM) chimeras were generated by lethal irradiation of C57BL/6J (CD45.2), TLR7^−/−^ (CD45.2), and C57BL/6J (CD45.1) recipient mice with a dose of 9 Gy for 409 s (160 kV, 25 mA) and reconstitution with intravenous injection of 4 × 10^6^ of Wt (C57BL/6J, CD45.1) or TLR7^−/−^ (CD45.2) BM cells. Mice were allowed to reconstitute for 7–8 weeks prior to analysis.

### Single Cell Suspension Preparation

For flow cytometric analysis of lymph nodes, tissues were digested in RPMI 1640 Glutamax medium supplemented with 100 U/ml Penicillin/Streptomycin, 1 mg/ml Collagenase D and 100 μg/ml DNase I for 30 min at 37°C. Single cell suspensions were prepared and a viable cell count was performed by trypan blue exclusion. For flow cytometric analysis of the foot skin, skin tissues were incubated in 2.5 mg/ml of Dispase II (Roche) solution prepared in RPMI + 5% FCS medium for 90 min at 37°C. Tissues were minced and digested in RPMI in the presence of 3 mg/ml Collagenase D and 100 μg/ml DNase I for 30 min at 37°C, followed by single cell suspension preparation.

### Flow Cytometry and Cell Sorting

All steps of flow cytometric staining were performed in FACS buffer containing PBS with 2% FCS and 2 mM EDTA, as described before (31). Acquisition and analysis of samples were performed using BD LSR II flow cytometer, and FlowJo software, respectively. Single stains and fluorescence minus one controls were used for accurate gating and compensation.

Cell sorting was performed using FACSAria (BD-Biosciences) at the Cell Sorting Core facility of the Hannover Medical School. The purity of the sorted cells was always >90%.

### Antibodies

All antibodies were purchased from eBioscience, except for anti-CD169 and anti-CD103 antibodies purchased from Biolegend. The following fluorochrome conjugated anti-mouse antibodies were used: CD11c (N418), B220 (RA3-6B2), CD11b (M1/70), CD169 (3D6.112), F4/80 (BM8), PDCA-1 (eBio927), Siglec-H (440c), EpCAM (G8.8), CD103 (2E7), MHC Class II (I-A/I-E) (M5/114.15.2), and Ly-6C (HK1.4).

### *In vivo* Imaging of VSV Infection in Mice

Hair on the lower abdominal area, hind limbs, and back of mice was shaved and epilated 1 day before infection. Mice were infected s.c. with 5 × 10^5^ pfu (for short term kinetics) or 5 × 10^6^ pfu (for survival experiments) of VSV-Luciferase. At corresponding time points p.i., mice were injected (i.p.) with 150 mg/kg of D-luciferin (Perkin-Elmer) in PBS, anesthetized with vaporized 2.5% isoflurane, and monitored using an IVIS Spectrum CT (PerkinElmer). Briefly, whole-body images were taken at a binning factor of 8 over 1–2 min for VSV-derived bioluminescence signal detection. For detection of three-dimensional bioluminescent signal, anesthetized mice were first scanned by computed tomography (CT) and viral bioluminescent signal was excited at 612 nm. Pseudo-color images displaying bioluminescence signal intensities in the organs were analyzed using the Living Image 4.3.1 software.

### Generation of Flt3-L Differentiated BM-Derived DC (BMDC) and *in vitro* Spin Inoculations

BMDC were generated in the presence of Flt3 ligand generated in-house as previously described (31). At day 9 of culture, cells were collected and FACS sorted as cDC (CD11c^+^ Siglec-H^−^) and pDC (CD11c^+^ Siglec-H^+^). Sorted cells were seeded at 5 × 10^4^/well density in 96-well flat bottom plates and infected with VSV-Luciferase (MOI 5) by spin infection (1,500 rpm for 30 min at 37°C). DC were incubated at 37°C, 5% CO_2_ till harvested and used for subsequent assays. For some experiments, sorted BM-derived cDC and pDC were infected with MOI 10 of VSV-eGFP by spin infection. Flt3-L producing CHO Flt3-L FLAG cells were generated by N. Nicola and kindly provided by Dr. K. Murphy (WEHI, Melbourne, Australia).

### Generation of LCCM-Differentiated BM-Derived Macrophages

BM cells isolated from femora and tibiae were seeded at a density of 5 × 10^6^ cells in 10 ml of RPMI 1640 Glutamax medium supplemented with L929 cell conditioned medium (LCCM, self-made) as a source of murine M-CSF and cultured for 7 days at 37°C, 5% CO_2_. On day 3, half of the medium in the plates was replenished with fresh RPMI containing LCCM. On day 7 of culture, cells were collected and seeded at 5 × 10^4^/well density in 96-well flat bottom plates. BM-macrophages were allowed to attach on the well surfaces overnight at 37°C, 5% CO_2_ and infected next day with VSV-Luciferase (MOI 5) by spin infection. LCCM producing L929 cell line was kindly provided by R. Lang (Universitätsklinikum Erlangen, Erlangen, Germany).

### Luciferase Assay

Replication of VSV-luciferase within the cells *in vitro* was determined by measuring the firefly luciferase activity in the cell lysates as described before (32). Briefly, following corresponding time points of infection, BM-derived cells were lysed in 35 μl of 1x Passive lysis buffer (Promega) and frozen down at −20°C overnight. Twenty microliters of cell lysate/sample was transferred into white 96-well plate. Assay buffer (25 mM glycylglycine, 15 mM MgSO4, 4 mM EGTA, 15 mM KPO4 pH 7.8, 1 mM DTT, 2 mM ATP) and substrate luciferin solution (25 mM glycylglycine and 200 μM luciferin, pH 8.0) were prepared in distilled water. Firefly luciferase activity was assessed for 1 s using a plate luminometer (Berthold Centro XS3 LB960 luminometer), with automatic injection of 72 μl of assay buffer and 40 μl of substrate per well. Measured relative light units (RLU) were plotted as a quantification of VSV replication in target cells.

### Virus Binding and Uptake Assay

BM-derived pDC were FACS-sorted from Flt3-L differentiated BMDC culture and 3 × 10^5^ cells were seeded per well. Cells were infected with MOI 5 of VSV-Indiana without spin infection and kept on ice for 1 h. For binding assay, cells on ice were washed with sterile PBS to remove the unbound viral particles and lysed in RLT buffer (Qiagen) for subsequent RNA isolation step. For uptake experiments, cells on ice were washed and cultured in fresh RPMI medium at 37°C, 5% CO_2_ for 30 min, or 2 h to allow uptake of VSV by BM-pDC. At corresponding time points, cells were lysed in RLT buffer following the washing step in PBS. The VSV amount bound on or taken up by the cells was determined by assessing *VSV-N* gene mRNA expression by RT-PCR.

### Depletion of Langerin^+^ DC in the Skin

Langerin-DTR:eGFP mice (25) were injected i.p. with 50 ng/gr of Diphtheria toxin (DT) prepared in PBS at least 48 h before VSV infection to deplete all Langerin^+^ DC. Some Langerin-DTR:eGFP mice only received 150 μl of PBS i.p. as non-depleted control group. Depletion efficiency was determined by assessing the frequency and number of Langerin^+^ cells in foot skin via flow cytometry.

### Histology

Cryostat sections (8 μm thick) of snap-frozen lymph nodes were air-dried, fixed in acetone for 10 min, and blocked with PBS containing 2% FCS. Sections were stained with the following anti-mouse antibodies in blocking solution for 45 min: monoclonal antibody to VSV glycoprotein (VSV-G, self-made), anti-CD169 (3D6.112, Acris Antibodies; 645608, R&D Systems), anti-CD45R (B220) (RA3-6B2, eBiosciences), and anti-F4/80 (BM8, eBiosciences). Imaging was performed on a KEYENCE BZ II analyzer fluorescence microscope.

### Enzyme-Linked Immunosorbent Assay

Type-I IFN levels in the supernatants of *in vitro* infected cells, in serum and organ homogenates of mice were determined using rat monoclonal anti-mouse IFN alpha antibody (clone RMMA-1), rabbit polyclonal anti-mouse IFN alpha antibody, and mouse IFN alpha A recombinant protein purchased from Pbl Interferon Source as described before (31).

### RNA Isolation

Total RNA from frozen organs were purified using TRIzol reagent (Life Technologies, Thermo Fisher Scientific) according to manufacturer's instructions. RNA isolation from FACS sorted pLN cells as well as *in vitro* generated BM-cDC, pDC, and macrophages was performed using Qiagen RNeasy Micro kit following manufacturer's instructions. RNAs were eluted in RNase-free water and stored either at −80°C or reverse transcribed in subsequent cDNA preparation step.

### Reverse Transcription and Real Time Quantitative PCR

Total RNA were transcribed into cDNA using random hexamer primers and SuperScript™ III Reverse Transcriptase system (Invitrogen). cDNAs were used for a SYBR green-based real-time quantitative PCR assay. Real time PCR amplification was performed with the LightCycler 480 system (Roche). Cycling conditions were 10 min at 95°C, followed by 40 cycles of 15 s (s) at 95°C, 30 s at 60°C, and 30 s at 72°C. The primer sequences used are as follows: *VSV-N* (F): 5′- TGATAGTACCGGAGGATTGACGAC-3′, (R): 5′-CCTTGCAGTGACATGACTGCTCTT-3′; *IFN-*β (F): 5′-CTTCTCCGTCATCTCCA TAGGG-3′, (R): 5′-CACAGCCCTCTCCATCAACT-3′, *TNF-*α (F): 5′-CATCTTCTCAAA ATTCGAGTGACAA-3′, (R): 5′-TGGAGTAGACAAGGTACAACCC-3′; *IL-12p40* (F): 5′-TCTTTGTTCGAATCCAGCGC-3′, (R) 5′-GGAACGCACCTTTCTGGTTACA-3′, *Usp18* (F): 5′-CCTGGAAGGATGTCCAGTGT-3′, (R): 5′-TTGAAATGCAGCAGACAAGG-3′; *TLR7* (F): 5′-CCTGTTCTACTGGGGTCCAA-3′, (R): 5′-GCCTCAAGGCTCAGAAGATG-3′ and β*-actin* (F): 5′-TGTTACCAACTGGGACGACA-3′, (R): 5′-GGGGTGTTGAAGGTCTCAAA-3′. Oligonucleotide primer pairs were synthesized by Eurofins MWG Operon (Ebersberg, Germany). PCR primers for IL-6 were purchased from SABiosciences.

### Statistical Analysis

All statistical analyses were performed using GraphPad Prism 6.0 software. The statistical difference among tested groups was analyzed using two-tailed *t*-test, Mann–Whitney test or Two-Way ANOVA (with Bonferroni post-test) and indicated in legends of corresponding figures. Unless otherwise stated, the data presented here were plotted as mean ± standard deviation. A *p*-value < 0.05 was considered significant and indicated by asterisk sign. ^*^p < 0.05, ^**^p < 0.01, ^***^p < 0.001, ^****^p < 0.0001.

## Results

### TLR7 Function Controls the Onset of VSV-Induced CNS Disease

**Figure 1:**
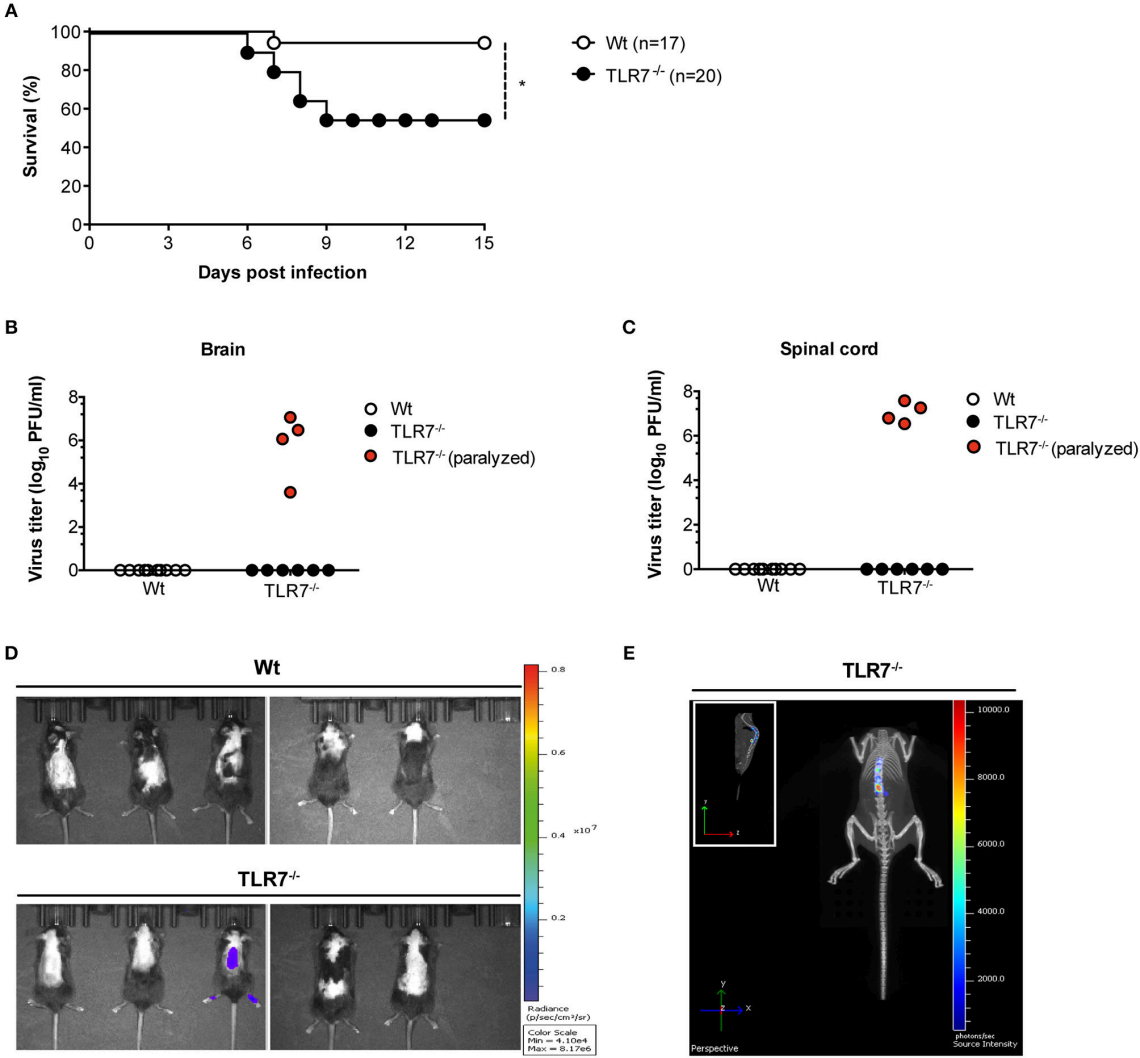
TLR7 plays an essential role in preventing fatal CNS invasion induced by peripheral VSV infection. **(A)** Survival curves of Wt (*n* = 17) and TLR7^−/−^ (*n* = 20) mice infected s.c. with 5 × 10^6^ pfu of VSV-Indiana. Shown here are pooled data of two independent experiments with similar results. **(B,C)** Viral burden in **(B)** brain and **(C)** spinal cord of Wt and TLR7^−/−^ mice sacrificed on day 11–15 p.i. or on the day of paralysis. **(D,E)** VSV dissemination into the CNS displayed as **(D)** viral bioluminescence alone or **(E)** an overlay with mouse computer tomography (CT) at day 10 post VSV-luciferase infection (*n* = 5 mice/group). Results in are representative data from two **(B,C,E)** or three **(D)** independent experiments showing similar results. The significance of differences between groups in **(A)** was analyzed by Log-Rank test. ^*^p < 0.05.

TLR7 and the downstream signaling protein MyD88 are key sensors of VSV, triggering robust production of type-1 IFN (9). After systemic infection, MyD88^−/−^ mice succumb to VSV-induced CNS disease (15). However, despite the loss of MyD88 function, VSV-induced type-I IFN production remains unaltered in these mice (15, 33) with reduced maintenance of neutralizing antibodies (33). Thus, the precise functional contribution of the TLR7/MyD88 pathway in regulating anti-VSV immunity remains controversial and deserves further clarification. Since VSV is an arthropod borne virus, we explored the spread of VSV from the skin, its entry site, to the CNS and investigated the function of TLR7 signaling in viral control. We first s.c. infected Wt and TLR7^−/−^ mice and compared their survival over the course of infection. After day 6 of infection, TLR7^−/−^ mice developed signs of hind limb paralysis and by day 9 post infection (p.i.), 40–50% had succumbed to infection as compared to Wt mice (Figure 1A). Closer examination of paralyzed TLR7^−/−^ mice revealed significantly higher viral loads in the brain and spinal cord than in non-paralyzed TLR7^−/−^ and Wt mice, where the virus was undetectable (Figures 1B,C). We next s.c. infected Wt and TLR7^−/−^ mice using a luciferase-expressing strain of VSV (VSV-luciferase) (29) and performed *in vivo* bioluminescence imaging to track the onset of neuroinvasion *in vivo*. By day 10 p.i., Wt mice had completely cleared the virus, while one in five TLR7^−/−^ mice developed hind limb paralysis and showed a strong bioluminescent signal in the spinal cord, which correlates with VSV infection (Figures 1D,E). Similarly, three in six MyD88^−/−^ mice displayed symptoms of neuroinvasion after day 10 of s.c. VSV-luciferase infection, and the virus was detectable in the brain and spinal cord of paralyzed MyD88^−/−^ mice using the *in vivo* imaging system (Supplementary Figure 1A) with computed tomography (Supplementary Figure 1B). Overall, our results show that similar to MyD88 deficiency, complete loss of TLR7 predisposes mice to increased susceptibility to VSV-induced neurovirulence after s.c. infection.

### Reduced VSV Titers in dLN of TLR7^−/−^ Mice Despite an Attenuated Proinflammatory Cytokine Response

**Figure 2:**
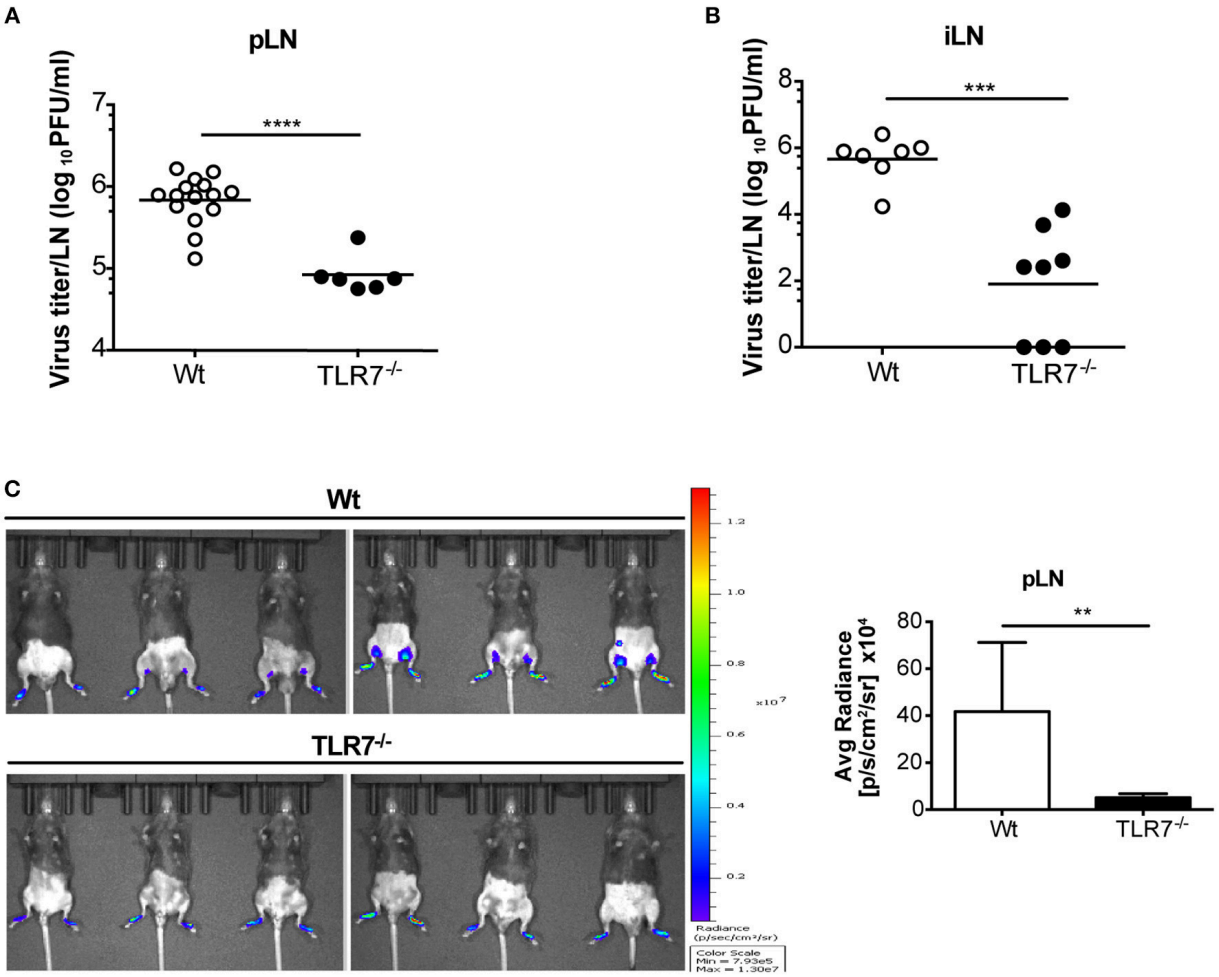
The absence of TLR7 reduces lymph-borne VSV infection in draining lymph nodes. **(A,B)** VSV titers in **(A)** pLN and **(B)** iLN at 12 h post s.c. VSV-Indiana (5 × 10^5^ pfu) infection. Results are **(A)** pooled from or **(B)** representative of two independent experiments (*n* = 3–8 mice/group /experiment). **(C)** Left: *In vivo* bioluminescence images of control and TLR7^−/−^ mice 12 h after s.c. 5 × 10^5^ pfu of VSV-luciferase infection. Right: Average radiance measured on pLN of mice shown in **(C)**. Data are from one of three individual experiments with similar results. The significance of differences between groups was analyzed by **(A,B)** two-tailed *t*-test or **(C)** Mann–Whitney test. ns, non-significant, ^**^p < 0.01, ^***^p < 0.001, ^****^p < 0.0001. Results are depicted as mean **(A,B)** or mean ± standard deviation **(C)**.

TLR7^−/−^ and MyD88^−/−^ mice developed hind-limb paralysis and succumbed to s.c. VSV infection (Figure 1D; Supplementary Figure 1A). Therefore, we next investigated if lack of TLR7/MyD88 signaling contributed to uncontrolled VSV infection in the skin dLN, which facilitates the entry of virus into peripheral nerves. To address this point, we infected Wt, TLR7^−/−^, and MyD88^−/−^ mice s.c. with Wt VSV via the foot skin and determined the virus concentration in the draining popliteal and inguinal LN at 12 h p.i. Counterintuitive to our hypothesis, both TLR7^−/−^ (Figure 2A) and MyD88^−/−^ (Supplementary Figure 1C) mice displayed significantly reduced virus titers in the popliteal LN (pLN). Similarly, inguinal LN (iLN) showed reduced infection in TLR7^−/−^ as compared to Wt mice (Figure 2B). We further visualized VSV infection *in vivo* by infecting mice with VSV-luciferase. After 12 h p.i., VSV was restricted to the foot skin in TLR7^−/−^, while the virus was detectable in the draining pLN of Wt mice (Figure 2C). Next, we compared the kinetics of infection in the pLN of Wt and TLR7^−/−^ mice. Mice were infected either with Wt VSV (Supplementary Figure 2A) or VSV-luciferase (Supplementary Figure 2B) and VSV load in the pLN was monitored from 0 h up to 12 h p.i. We observed that from 3–6 h onwards VSV infection was significantly reduced in the pLN of TLR7^−/−^ as compared to Wt mice (Supplementary Figures 2A,B). Furthermore, we tested whether the difference in LN infection between Wt and TLR7^−/−^ mice was restricted to the early phase until 12 h p.i. by performing a kinetic analysis in dLN at later time points. We observed a similar but less pronounced reduction in viral titers in the pLN and in the iLN of TLR7^−/−^ mice at 48 h p.i., while by 72 h p.i. the virus was close to the detection limit and mostly undetectable in the dLN (Supplementary Figures 2C,D). Simultaneously, in the spleen a low but comparable VSV load was observed in Wt and TLR7^−/−^ mice at 12 h p.i. (Supplementary Figure 2E). Next, we investigated whether the effect of TLR7 on VSV dissemination was dependent on the site of infection. To this aim, we infected mice with VSV intra-dermally (i.d.) in the ear skin and systemically via the intravenous (i.v.) route. Similar to our results obtained after s.c. infection, at 12 h p.i. TLR7^−/−^ exhibited reduced VSV loads as compared to Wt mice in the ear dLN after i.d. and in the spleen after i.v. infection (Supplementary Figures 2F,G). Furthermore, we investigated the role of TRIF and RLR pathways, known to recognize RNA viruses (34, 35), in peripheral VSV infection. Our preliminary results showed that in contrast to TLR7^−/−^ mice, viral loads in dLN of TRIF^−/−^ (36), and CARDIF^−/−^ (37) mice were significantly higher than in Wt mice suggesting that both pathways contribute in controlling the s.c. VSV infection (Supplementary Figure 2H).

**Figure 3:**
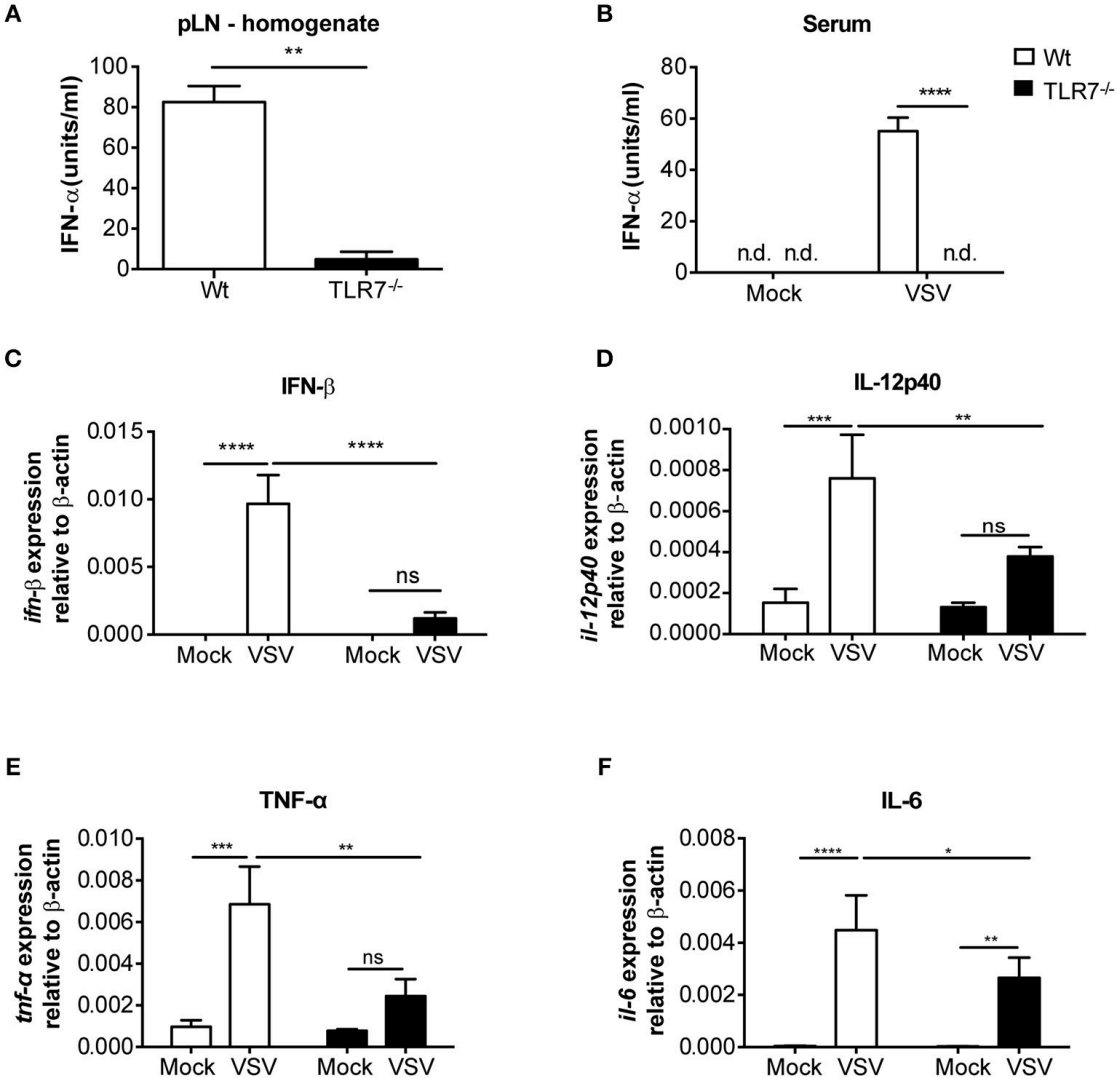
VSV-induced cytokine responses are reduced in dLNs of TLR7^−/−^ mice. **(A,B)** IFN-α concentrations in **(A)** pLN homogenate and **(B)** sera of mice either treated with PBS (Mock) or infected with VSV-Indiana s.c. at 12 h p.i. **(C–F)** Expression of **(C)** *IFN-*β, **(D)** *IL-12p40*, **(E)** *TNF-*α, and **(F)** *IL-6* mRNA in total pLN cells of Wt and TLR7^−/−^ mice at 12 h post s.c. PBS (Mock) treatment or 5 × 10^5^ pfu of VSV-Indiana infection (*n* = 3–5 mice/group/experiment). Results were assessed relative to expression of housekeeping gene β*-actin* and depicted as mean ± standard deviation. Data shown in **(A–F)** are from one of three individual experiments with similar results. The significance of differences between groups was analyzed by **(A)** Mann–Whitney test or **(B–F)** Two-Way ANOVA. ns, non-significant. ^*^p < 0.05, ^**^p < 0.01, ^***^p < 0.001, ****p < 0.0001. Results are depicted as mean ± standard deviation.

We next examined the local cytokine milieu in the dLN of VSV infected TLR7^−/−^ mice in which VSV infection was reduced. Our results revealed that, as expected, due to the lack of sensing via TLR7, IFN-α levels in the pLN were significantly diminished (Figure 3A). A similar reduction in IFN-α was observed in the serum after 12 h p.i. (Figure 3B). Recently, it has been demonstrated that TLR7-mediated IFN-β derived from astrocytes blocks VSV replication in the brain (38). Hence, we additionally measured IFN-β mRNA transcript levels in the dLN of VSV infected TLR7^−/−^ mice. We observed that induction of IFN-β expression after infection was also significantly reduced in TLR7^−/−^ mice as compared to Wt controls (Figure 3C). To determine if reduced VSV loads in TLR7^−/−^ mice, despite an attenuated type I IFN response, was due to cell intrinsic inhibitory factors, we next measured Usp18 gene expression in mock treated or VSV infected Wt and TLR7^−/−^ pLN. Absence of Usp18 has been suggested to lead to hyperphosphorylation of the type-I IFN receptor (16) enabling an increased antiviral state of cells by sustained signaling even in the presence of reduced type-I IFN levels (39). Unlike these findings, we observed comparable levels of Usp18 mRNA expression in Wt and TLR7^−/−^ dLN after s.c. VSV infection (Supplementary Figure 3A). Similarly, we examined the gene expression levels of Mx2 and Viperin, IFN-stimulated genes known to control VSV infection (40, 41) in dLN of mock treated or VSV infected Wt and TLR7^−/−^ mice. Our results demonstrated that Mx2 expression is similarly upregulated in control and TLR7 deficient pLN after VSV infection (Supplementary Figure 3B). On the other hand, VSV-induced an increase in Viperin expression which was significantly impaired in TLR7^−/−^ pLN compared to Wt mice (Supplementary Figure 3C). These data suggest that reduced VSV infection in draining LN in the absence of TLR7 is not a result of excessive antiviral immune responses. We next measured the expression of an autophagy gene, Atg5, known to negatively regulate antiviral immune responses (42) in Wt, and TLR7^−/−^ pLN and found that Atg5 expression is reduced at a comparable level in pLN of Wt and TLR7^−/−^ mice after infection (Supplementary Figure 3D). Aside from type-I IFN production, IL-12p40 (Figure 3D), TNF-α (Figure 3E), and IL-6 (Figure 3F) gene expression levels were also reduced after VSV infection in TLR7^−/−^ mice as compared to infected Wt mice. Overall, our results indicate that TLR7^−/−^ mice develop reduced VSV infection in the dLN despite compromised antiviral and pro-inflammatory cytokine responses.

### TLR7 Signaling Controls VSV Infection in the Foot Skin

**Figure 4:**
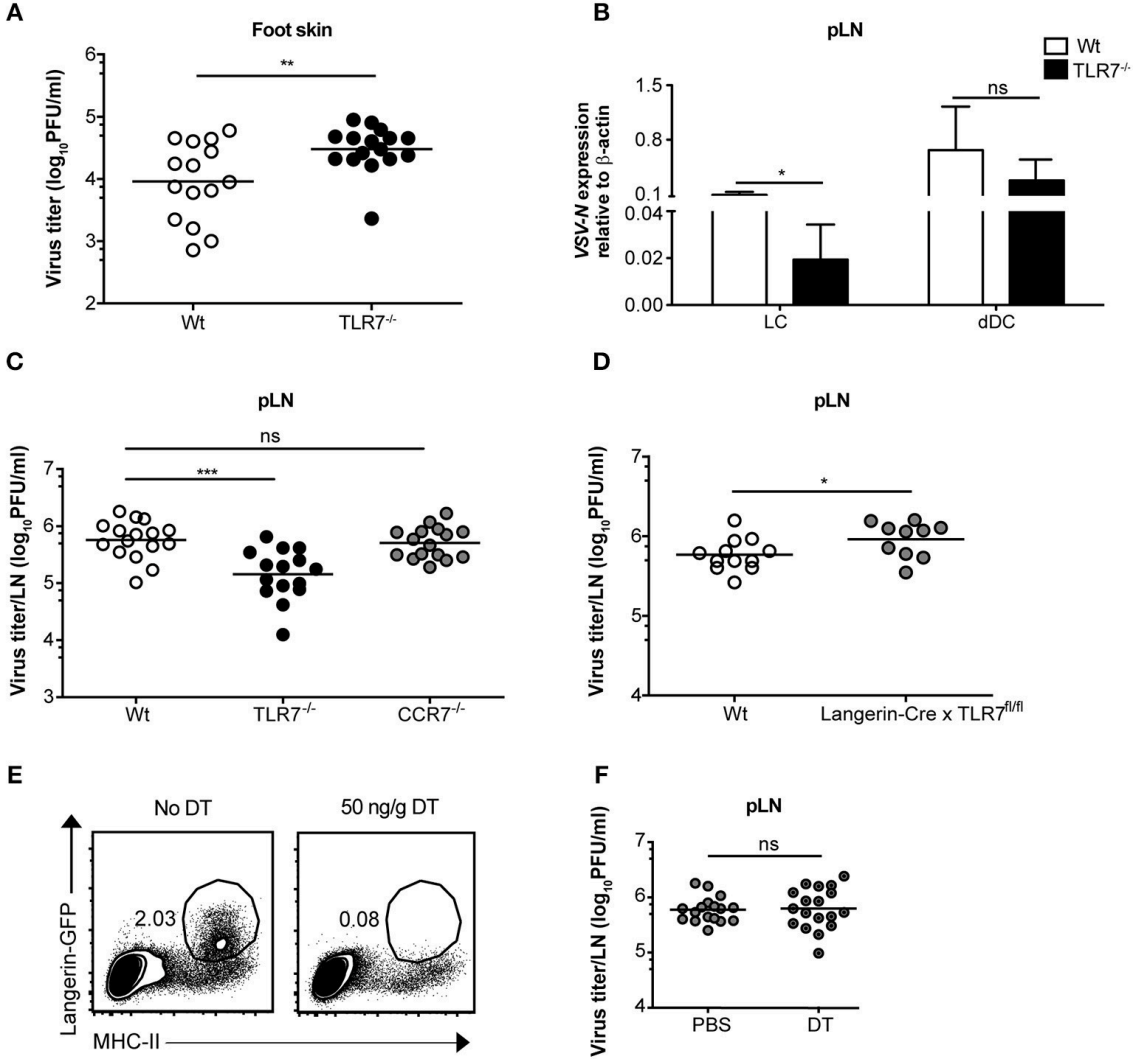
TLR7 function in skin cells is important to control VSV infection in the foot skin but dispensable for development of early VSV infection in pLN. **(A)** Viral titers in foot skin of control and TLR7^−/−^ mice after infection at 12 h with 5 × 10^5^ pfu of VSV-Indiana (*n* = 3–5 mice/group/ experiment). **(B)** Expression of *VSV-N* mRNA in Langerhans cells (LC) and Langerin^+^ dermal dendritic cells (dDC) which were FACS-sorted from pLN of VSV-Indiana infected Wt and TLR7^−/−^ mice at 12 h p.i. **(C)** VSV titers in pLN of mice 12 h after s.c. VSV-Indiana infection (*n* = 2–8 mice/group/experiment). **(D)** Viral titers in pLN of control mice and conditional TLR7 knockout mice lacking TLR7 function in Langerin^+^ cells (Langerin-Cre x TLR7^fl/fl^ mice), *n* = 4–6 mice per group. **(E)** Left panel: Representative FACS plots presenting the efficiency of DT treatment (i.p.) to deplete Langerin-GFP^+^ cells in the foot skin of Langerin-DTR mice at 48 h post single dose injection (50 ng/g) of DT. Langerin-DTR mice in “No DT” group were treated (i.p.) with sterile PBS. Right panel: VSV titers assessed at 12 h p.i. in pLN of Langerin-DTR mice treated either with PBS or 50 ng/g of DT 48 h prior to VSV-Indiana infection (*n* = 3–6 mice/group/experiment). Results show the pooled data from **(D)** two, **(C)** three or **(A,B,E)** four independent experiments with similar results. Data are depicted as **(A,C–E)** mean or **(B)** mean ± standard deviation. The significance of differences between groups was analyzed by **(A,C–E)** two-tailed *t*-test or **(B)** Two-Way ANOVA. ns, non-significant. ^*^p < 0.05, ^**^p < 0.01, ^***^p < 0.001.

Since we observed an essential function of TLR7 in the development of early VSV infection in dLN (Figures 2A,B), we queried whether this is also required at the very first site of infection. To test this hypothesis, we compared viral infection in the foot skin (site of virus administration) of Wt and TLR7^−/−^ mice. In contrast to our hypothesis, 12 h p.i. TLR7^−/−^ mice harbored a modestly higher viral load (Figure 4A) with impaired type-I IFN responses (Supplementary Figure 4A) in the skin as compared to Wt. Similarly, VSV-luciferase infection assessed by *in vivo* imaging was restricted to the foot skin in TLR7^−/−^ mice (Figure 2C). Thus, we determined whether the increased and reduced viral loads in the foot skin and the dLN, respectively were a consequence of impaired cell dependent VSV transport from the skin to the dLN. To test this, we *ex viv*o FACS-sorted skin-derived Langerhans cells (LC) and Langerin^+^ dermal DC (dDC) at 12 h post s.c. VSV infection (Supplementary Figure 4B) and measured *VSV-N* mRNA expression in the purified dLN populations. We observed that migratory dDC displayed a high level of *VSV-N* mRNA expression, followed by LC (Figure 4B). Furthermore, TLR7^−/−^ migratory DC in the draining pLN showed reduced *VSV-N* expression as compared to their Wt counterparts (Figure 4B). Thus, our results suggest that skin-derived DC may transport VSV inoculated into the foot skin to dLN and that TLR7 signaling may contribute to this function of antigen transport. CCR7 is an important pro-migratory chemokine receptor for leukocyte homing to the LN (43). Hence, we investigated whether CCR7-dependent mechanisms controlled VSV transport from the skin to the dLN and contributed to the reduced viral load in the dLN of TLR7^−/−^ mice. To test this hypothesis, we examined viral titers in the dLN of VSV infected CCR7^−/−^ mice. However, VSV loads in the pLN remained unaffected in the absence of CCR7 signaling as compared to Wt mice (Figure 4C). Furthermore, we found that CXCR4, another important pro-migratory chemokine receptor for dermal and epidermal DC mobilizing to the dLN (44), was also redundant for the development of early VSV infection in the dLN. Inhibition of CXCR4 activity by a single s.c. injection of AMD3100 (100 μg/foot skin), a selective CXCR4 antagonist, administered 2 h prior to infection failed to alter viral loads in the pLN of Wt and TLR7^−/−^ mice as compared to vehicle treated littermate controls (Supplementary Figure 4C). Next, we sought to determine the role of TLR7 in skin DC (LC and Langerin^+^ dDC) in VSV antigen transport from the skin to the dLN. For this purpose, we generated a novel TLR7^fl/fl^ transgenic mouse line (Supplementary Figure 4D), in which exon 3 (functional coding exon) of the *Tlr7* gene was flanked by *loxP* sites. To delete TLR7 function in Langerin^+^ skin DC, we crossed TLR7^fl/fl^ to Langerin-Cre (24) mice (further denoted as Langerin-Cre x TLR7^fl/fl^). We s.c. infected Wt (Cre negative) and Langerin-Cre x TLR7^fl/fl^ littermates, and measured the virus load in pLN at 12 h p.i. Our results indicate that loss of TLR7 function in Langerin^+^ DC moderately enhanced VSV infection in the pLN of Langerin-Cre x TLR7^fl/fl^ mice (Figure 4D). To demarcate precisely the contribution of skin DC in the establishment of early VSV infection in dLN, we next set out to deplete all Langerin^+^ cells in the skin prior to infection by administering DT to Langerin-DTR:eGFP mice (25). We observed that although efficient ablation of Langerin^+^ cells in the foot skin was achieved (Figure 4E, left panels), viral titers in the dLN remained unaltered (Figure 4E, right panel). Taken together, our results demonstrate that differences in viral load observed in the pLN of VSV-infected TLR7^−/−^ mice are independent of skin DC migration.

### TLR7 Deficiency Attenuates VSV Replication *in vitro*

**Figure 5:**
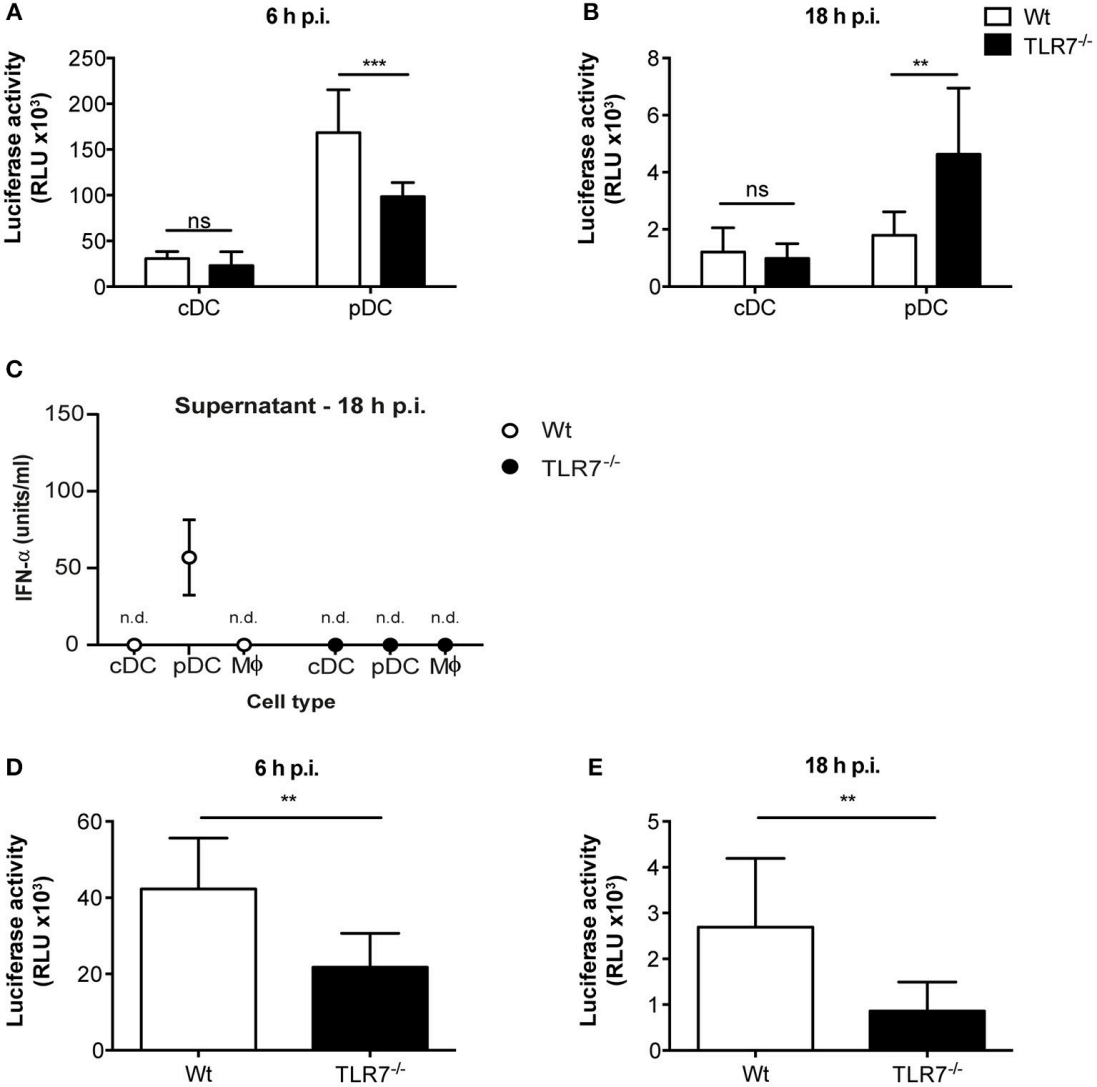
TLR7 is required for efficient VSV replication in BM-derived pDC and macrophages *in vitro*. **(A,B)** Luciferase expression, indicator of VSV-luciferase (MOI 5) infection, in Wt and TLR7^−/−^ BM-derived Flt3-L generated and FACS-sorted cDC and pDC at **(A)** 6 h and **(B)** 18 h p.i. assessed by firefly luminescence assay. Data are from one of three individual experiments with similar results. **(C)** IFN-α concentration in the supernatants of *in vitro* generated BM-derived cDC, pDC, and macrophages infected with VSV-luciferase at MOI 5 for 18 h *in vitro*. Results are depicted as mean ± standard deviation and show the pooled data from three independent experiments with similar results. n.d: not-detected **(D,E)** Luciferase expression in Wt and TLR7^−/−^ BM-derived LCCM generated macrophages at **(D)** 6 h and **(E)** 18 h post VSV-luciferase infection (MOI 5) assessed by firefly luminescence assay. Data are from one of three individual experiments with similar results. Results are depicted as mean ± standard deviation and significance of differences between groups was analyzed by **(A,B)** Two-Way ANOVA or **(D,E)** Mann–Whitney test. ns, non-significant. ^**^p < 0.01, ^***^p < 0.001.

Given that reduction of VSV loads in the dLN of TLR7^−/−^ mice was not a consequence of impaired DC migration, we next sought to elucidate whether virus replication was attenuated in the absence of TLR7 function. To test this hypothesis, we first infected Flt3-L differentiated bone marrow (BM)-derived pDC and conventional DC (cDC) with VSV-luciferase and performed a kinetics of luciferase expression *in vitro*. We observed that VSV-luciferase activity in TLR7^−/−^ pDC was significantly reduced compared to Wt pDC after 6 h of infection (Figure 5A). Simultaneously, Wt and TLR7^−/−^ cDC showed comparable VSV-luciferase expression (Figure 5A). Moreover, we infected BM-derived pDC with Wt VSV at a MOI of 5 to quantify the infectious VSV particles within these cells. Our results indicate that, in line with attenuated luciferase activity, TLR7^−/−^ pDC contained significantly fewer VSV particles as compared to Wt cells at 6 h p.i. (Supplementary Figure 5A), suggesting that TLR7^−/−^ pDC were less susceptible to VSV infection. In contrast, at 18 h p.i. VSV-luciferase expression was increased in TLR7^−/−^ pDC as compared to Wt pDC while expression levels in cDC were still comparable between the two groups (Figure 5B). We next determined whether robust IFN-α production from Wt pDC at 18 h p.i. blocked VSV replication in these cells, in contrast to TLR7^−/−^ pDC unable to produce IFN-α in response to VSV (9). Comparison of IFN-α levels in the supernatants of VSV-infected Wt and TLR7^−/−^ BM-derived cell types at 6 and 18 h p.i. revealed that Wt and TLR7^−/−^ pDC with strong VSV-luciferase expression at 6 h p.i. both failed to produce detectable IFN-α at this time point of infection (data not shown). On the other hand, while VSV-infected TLR7^−/−^ pDC remained unresponsive after 18 h, Wt pDC produced copious amounts of IFN-α at this later time point (Figure 5C). Next, we examined whether the differential kinetics between TLR7^−/−^ and Wt pDC was a consequence of virus replication and not mere attachment or uptake. For this purpose, we assessed the VSV amounts in infected pDC either incubated with Wt VSV on ice (to examine binding) or at 37°C for 30 min or 2 h (to determine internalization) by qPCR. Consistent with TLR7 expression being restricted to intracellular vesicles (45), our results showed that TLR7 deficient pDC possess an equal capacity for viral binding and uptake as their Wt counterparts (Supplementary Figure 5B). Furthermore, the reduced expression of VSV-driven luciferase was not a consequence of poor cell viability of TLR7^−/−^ cells, as the frequencies of live cDC and pDC of TLR7^−/−^ and Wt groups with or without VSV infection were comparable at 6 and 18h p.i. (Supplementary Figures 5C,D). Additionally, we examined VSV infection in *in vitro* generated BM-derived macrophages. Similar to what we observed with pDC, at 6 h p.i. VSV-luciferase expression was significantly reduced in TLR7^−/−^ macrophages as compared to Wt cells (Figure 5D). Interestingly, unlike in pDC, the reduction in luciferase levels in TLR7^−/−^ macrophages was still observed at 18 h p.i. (Figure 5E), while no IFN-α production was detectable in the supernatants of both Wt and TLR7^−/−^ BM-derived macrophages at this time point (Figure 5C). Next, we investigated whether different levels of TLR7 expression in BM-derived cDC, pDC, and macrophages resulted in the discrepancy of TLR7 contribution to VSV replication within these cells. By measuring *TLR7* mRNA transcript levels, we demonstrated that TLR7 expression levels are higher in both productively infected BM-derived macrophages and pDC in contrast to less infected cDC (Supplementary Figure 5E). In conclusion, our results show that intrinsic TLR7 is required for efficient early VSV replication *in vitro*.

### TLR7 Function in Both Hematopoietic and Non-hematopoietic Compartments Contributes to VSV Infection in the dLN

**Figure 6:**
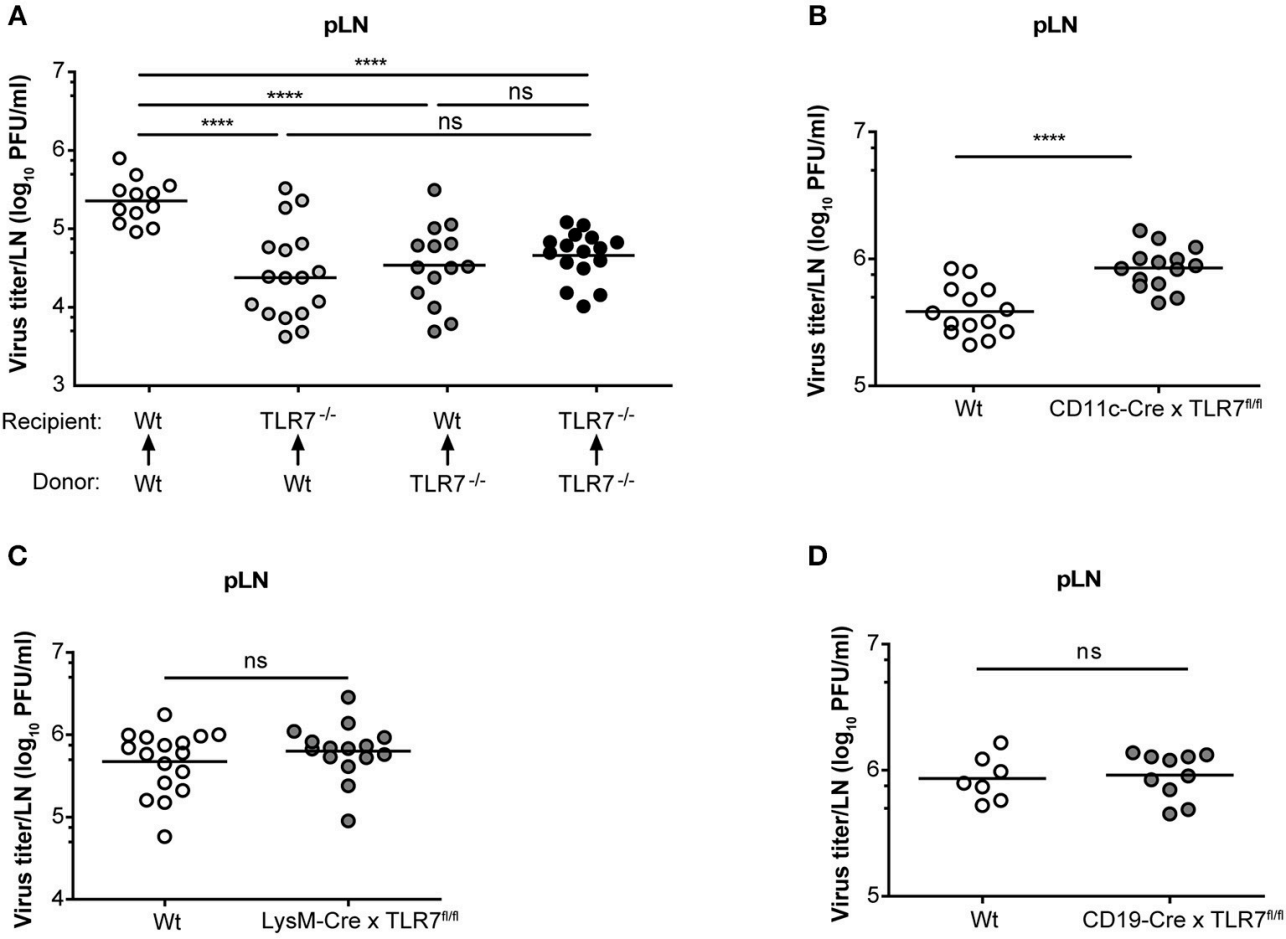
TLR7 in hematopoietic and non-hematopoietic cells synergize to promote early VSV infection in pLN. **(A)** VSV titers in pLN of lethally irradiated Wt and TLR7^−/−^ mice that were reconstituted with Wt or TLR7^−/−^ BM cells 7–8 weeks prior to s.c. VSV-Indiana infection (5 × 10^5^ pfu). Results are pooled from three experiments where *n* = 2–7 mice/group/experiment were used. **(B–D)** Viral titers in pLN of conditional TLR7 knockout mice deficient for TLR7 signaling in **(B)** CD11c^+^, **(C)** Lysozyme M^+^, or **(D)** CD19^+^ cells 12 h after VSV-Indiana infection. Results show mean values of pooled data from **(D)** two or **(B,C)** three experiments (*n* = 2–8 mice/group/experiment). ns, non-significant. ^****^p < 0.0001 (two-tailed *t*-test).

Mice lacking the interferon-α/β receptor (IFNAR) on stromal cells display slowly ascending CNS pathology, while SCS macrophage-derived type-I IFN protects mice from VSV-induced neurovirulence (14). Hence, we next compartmentalized the contribution of TLR7 function in non-hematopoietic and hematopoietic cells in regulating VSV replication in the dLN of mice. To address this point, we generated TLR7^−/−^ BM chimeras that either lacked TLR7 in the hematopoietic or non-hematopoietic compartment or in both. As controls, we included Wt chimeric mice with TLR7 function present in both compartments (Supplementary Figure 6A). Following 7–8 weeks of reconstitution with donor BM cells, mice were infected s.c. with VSV and the role of TLR7 in both hematopoietic and non-hematopoietic compartments in influencing viral load in dLN was evaluated. Our results indicated that TLR7 function in both compartments contributed to the establishment of VSV infection in pLN at 12 h p.i. (Figure 6A). Furthermore, local type-I IFN production was mainly triggered by hematopoietic cell-derived TLR7 signaling in pLN against lymph-borne VSV infection (Supplementary Figure 6B). Since the loss of TLR7 in either compartment reduced VSV loads in the pLN, we sought to dissect the cell-specific role of TLR7 in controlling viral replication *in vivo* using our novel TLR7^fl/fl^ transgenic mouse line. To delete TLR7 function in individual cell populations, we crossed TLR7^fl/fl^ mice to Cre-expressing lines under the control of CD11c (CD11c-Cre × TLR7^fl/fl^), Lysozyme-M (LysM-Cre × TLR7^fl/fl^), CD19 (CD19-Cre × TLR7^fl/fl^), and Podoplanin (Pdpn-Cre × TLR7^fl/fl^) promoter activity (the respective deletion efficiencies are depicted in Supplementary Figures 6C–E). Our results revealed that in contrast to TLR7^−/−^ mice, viral loads in the dLN of CD11c-Cre x TLR7^fl/fl^ mice were significantly higher than in Wt (Figure 6B). Interestingly, CD11c-Cre × TLR7^fl/fl^ mice and TLR7^−/−^ mice had comparably impaired IFN-α levels in dLN, compared to Wt controls (Supplementary Figure 7A). Additionally, deletion of TLR7 in CD11c^+^ cells did not affect the viral load in the foot skin assessed by standard plaque assay (Supplementary Figure 7B) and *in vivo* imaging system (Supplementary Figure 7C). Mice lacking TLR7 function in CD11c^+^ cells also failed to show increased susceptibility to VSV-induced CNS disease as compared to littermate controls (Supplementary Figure 7D). In contrast, VSV load remained unaltered in pLN (Figure 6C) and foot skin (Supplementary Figure 7E) of mice deficient in TLR7 function in LysM^+^ macrophages/neutrophils. Similarly, our results revealed that TLR7 in CD19^+^ B cells (Figure 6D) and Pdpn^+^ stromal cells (Supplementary Figure 7F) is dispensable to control peripheral VSV infection. In conclusion, complete loss of TLR7 function strongly reduced VSV replication in the draining pLN at 12 h p.i. and both hematopoietic and non-hematopoietic compartments contributed to this phenotype. Cre-mediated cell-specific loss of TLR7 function in CD11c^+^ myeloid cells, CD19^+^ B cells, LysM^+^ macrophages/neutrophils, and Pdpn^+^ stromal cells failed to mimic the phenotype of reduced VSV infection in the dLN observed in total TLR7^−/−^ mice.

### TLR7 Is a Vital Host Factor That Promotes SCS Macrophage Infection *in vivo*

**Figure 7:**
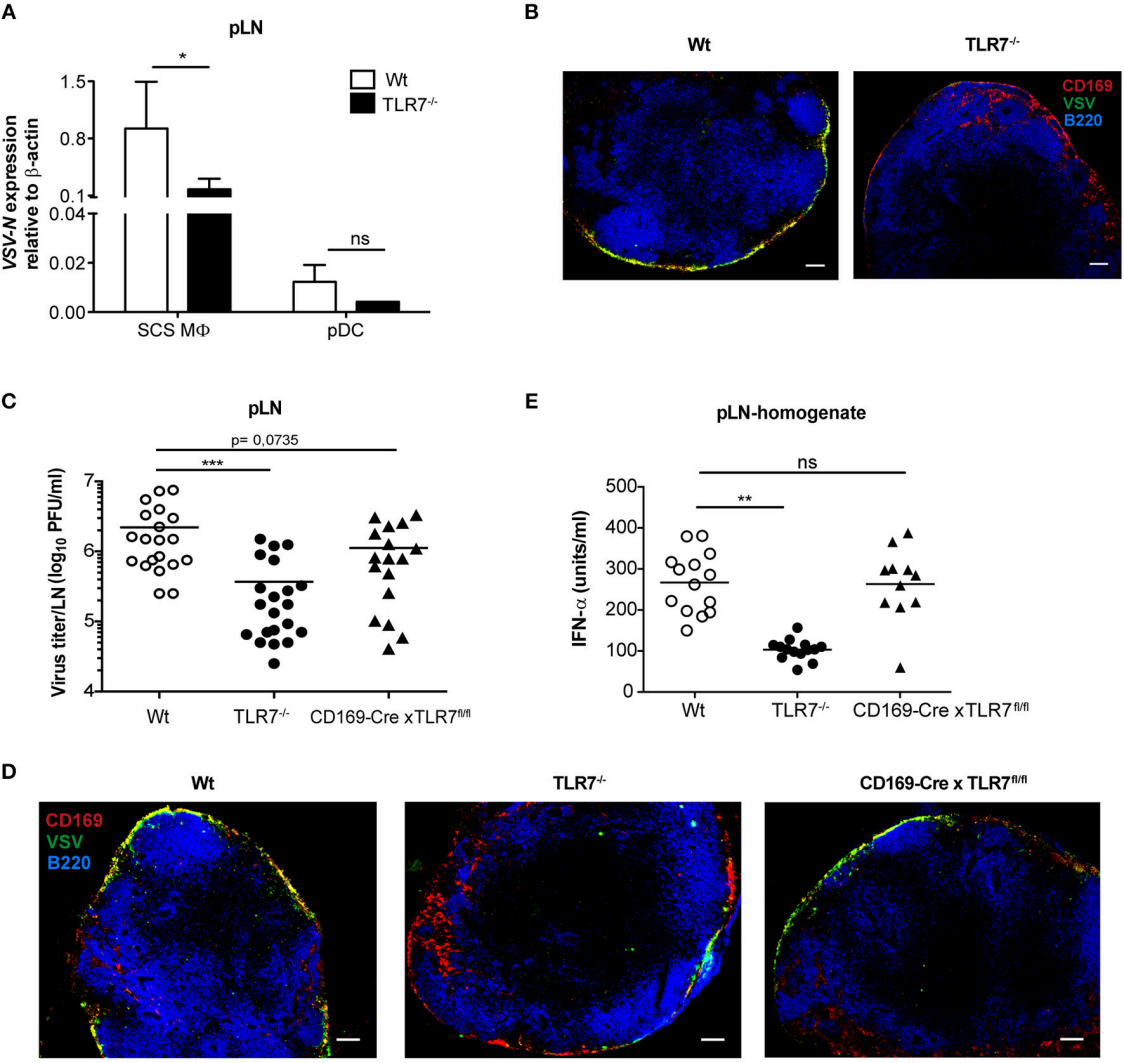
TLR7 serves as a host factor driving early VSV replication in CD169^+^ SCS macrophages in draining pLN. **(A)** Expression of *VSV-N* mRNA in CD169^+^ SCS macrophages (SCS MΦ) and plasmacytoid dendritic cells (pDC) which were FACS-sorted from pLN of VSV-Indiana infected Wt and TLR7^−/−^ mice at 12 h p.i. Results show the pooled data from four independent experiments with similar results. **(B)** Representative fluorescent microscopy images (10x) of pLN of Wt and TLR7^−/−^ mice 12 h after VSV-Indiana (5 × 10^6^ pfu) infection. Scale bars reflect 100 μm. Results are representative of two independent experiments (*n* = 2–3 mice/group/experiment). **(C)** Viral titers in pLN of Wt, TLR7^−/−^, and CD169-Cre × TLR7^fl/fl^ mice at 12 h p.i. (*n* = 2–5 mice per group). Results show the pooled data from five independent experiments with similar results. **(D)** Representative fluorescent microscopy images (10x) of pLNs of Wt (CD169-Cre negative littermate), TLR7^−/−^ and CD169-Cre × TLR7^fl/fl^ mice at 12 h post VSV-Indiana (5 × 10^6^ pfu) infection. Scale bars reflect 100 μm. Results are representative of two independent experiments (*n* = 2–3 mice/group/experiment). **(E)** IFN-α concentrations in pLN homogenate of mice infected with VSV-Indiana s.c. at 12 h p.i. (*n* = 2–5 mice per group). Results show the pooled data from four independent experiments with similar results. Data are depicted as **(C,E)** mean or **(A)** mean ± standard deviation. The significance of differences between groups was analyzed by **(A)** Two-Way ANOVA or **(C,E)** two-tailed *t*-test. ns, non-significant. ^*^p < 0.05, ^**^p < 0.01, ^***^p < 0.001.

Given that lymph-borne VSV primarily replicates in CD169^+^ macrophages lining the SCS layer of dLN (46), we sought to investigate VSV replication in SCS macrophages of Wt and TLR7^−/−^ pLN. For this purpose, we first *ex viv*o sorted pLN resident SCS macrophages and pDC at 12 h post s.c. VSV infection (Supplementary Figure 4B) and measured *VSV-N* mRNA expression in these cells. In line with the findings of Iannacone et al. (14), we observed that SCS macrophages express high levels of *VSV-N*, while pDC show very low viral gene expression (Figure 7A). Interestingly, lack of TLR7 resulted in a significant reduction in *VSV-N* expression in SCS macrophages (Figure 7A). Additionally, *TLR7* expression was upregulated in highly infected SCS macrophages but not in pDC at 12 h p.i. (Supplementary Figure 8A). Furthermore, we assessed VSV infection in SCS macrophages in pLN of Wt and TLR7^−/−^ mice using immunofluorescence microscopy. Consistent with diminished *VSV-N* mRNA levels, we observed that TLR7 deficiency induced a remarkable reduction in VSV protein expression in CD169^+^ cells lining the SCS layer (Figure 7B), suggesting that absence of TLR7 drastically attenuates VSV replication in SCS macrophages *in vivo*. We next set out to determine whether the reduced VSV protein expression in CD169^+^ cells was due to a compromised SCS macrophage layer in TLR7^−/−^ mice. To this end, we compared the LN architecture between mock treated Wt and TLR7^−/−^ mice and demonstrated that TLR7^−/−^ pLN displayed an architecture similar to Wt pLN (Supplementary Figure 8B). We further compared the cellularity and distribution of SCS macrophages and different immune cells between Wt and TLR7^−/−^ LN. Although the frequency of SCS macrophages was significantly reduced (Supplementary Figure 8C), the overall cell number of SCS macrophages and other LN immune cells between Wt and TLR7^−/−^ mice remained unchanged (Supplementary Figures 8D–F).

In line with our TLR7 expression data (Supplementary Figure 6D), Chtanova et al. reported that SCS macrophages were poorly targeted using the LysM specific promoter (47). To this end, cell-specific deletion of TLR7 in LysM^+^ cells did not cause a similar reduction in VSV titers in dLN as in TLR7^−/−^ mice (Figure 6C). Therefore, we next crossed our TLR7^fl/fl^ mice to mice expressing Cre recombinase under control of the CD169 promoter (27) (further denoted as CD169-Cre x TLR7^fl/fl^). This results in selective deletion of TLR7 signaling only in CD169^+^ cells, i.e., in SCS and medullary macrophages in dLN. CD169-Cre x TLR7^fl/fl^ mice showed a trend of reduced VSV infection in dLN as compared to Wt mice (*p* = 0.0735) although the reduction was less pronounced compared to TLR7^−/−^ mice (Figure 7C). Accordingly, VSV protein expression in CD169^+^ cells lining the SCS layer was only moderately impaired as depicted in representative microscopy pictures of pLN of CD169-Cre x TLR7^fl/fl^ mice (Figure 7D).

Given that loss of TLR7 function in CD169^+^ cells reduced VSV infection in the dLN to a lesser degree than in TLR7^−/−^ mice, we interrogated the targeting efficiency of CD169-Cre promoter for TLR7 in SCS macrophages of these conditional TLR7 knockout mice. We found that expression of *TLR7* gene was reduced by ~30% in SCS macrophages of CD169-Cre x TLR7^fl/fl^ mice (Supplementary Figure 9). Iannacone et al. reported that actively infected SCS macrophages are the main driving force of type-I IFN production in dLN of subcutaneously infected mice; producing local type-I IFN as well as stimulating pDC relocalization from the T cell zone to SCS where the viral antigens can be sensed by pDC via TLR7 for further type-I IFN production (14). Interestingly, although TLR7 deficiency in CD169^+^ cells partially impaired VSV infection of SCS macrophages, local IFN-α production in pLN of VSV-infected CD169-Cre x TLR7^fl/fl^ mice was not compromised (Figure 7E). Overall, our results establish that TLR7 regulates the unique permissiveness of SCS macrophages in dLN to facilitate early virus replication after lymph-borne VSV infection.

## Discussion

**Figure 8:**
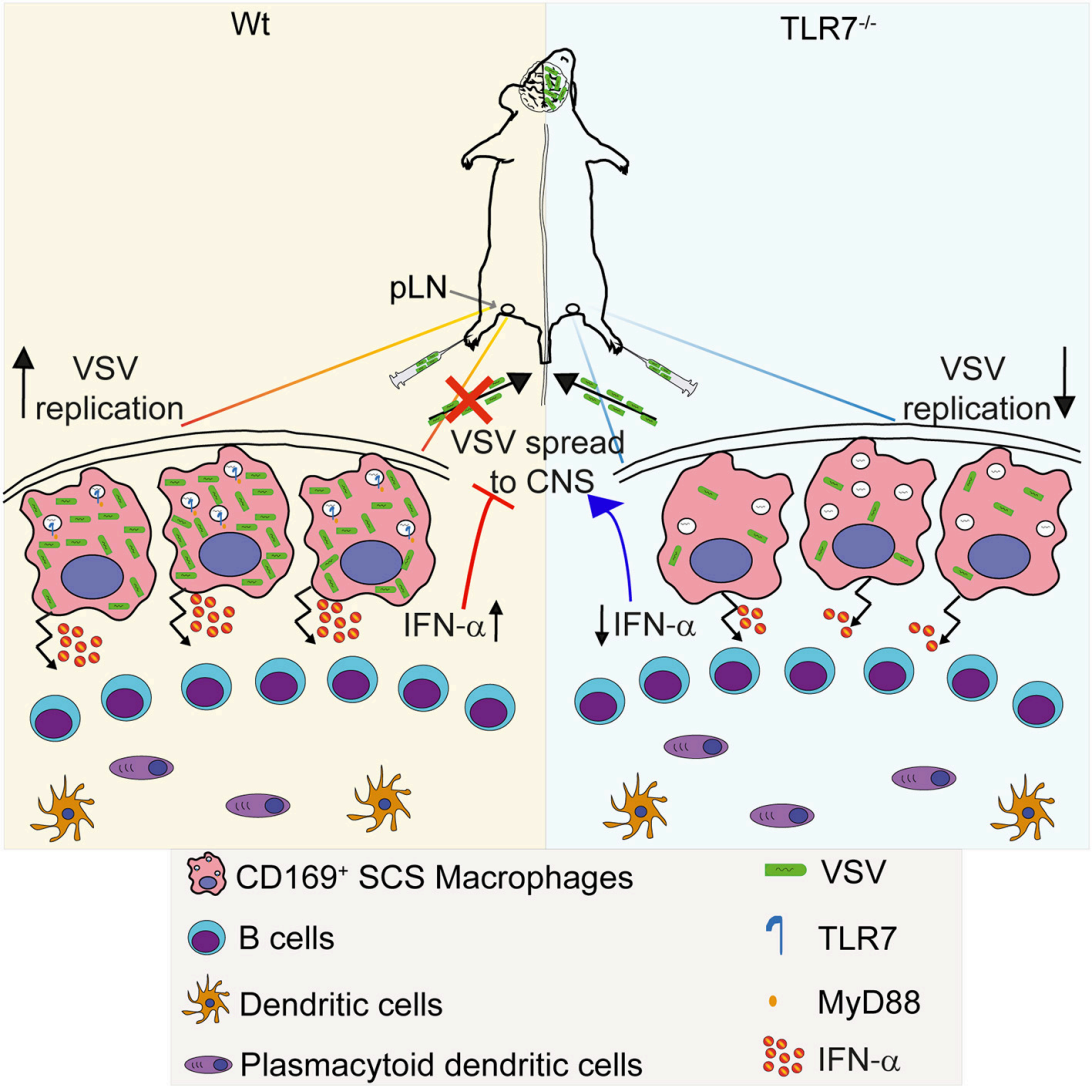
Graphical abstract. **(Left)** VSV injected subcutaneously via foot of mice reaches to draining pLN where the viral particles are captured by CD169^+^ macrophages lining the subcapsular sinus (SCS) layer. Virus is recognized via TLR7 and promptly replicates in SCS macrophages due to their low type I IFN responsiveness and low levels of lysosomal enzymes. Replicating virus activates SCS macrophages which produce type-I IFN *in situ* and simultaneously trigger migration of pDC toward SCS layer where pDC encounter VSV and produce additional type-I IFN. High concentrations of type-I IFN prevent entry of virus in peripheral nerves. **(Right)** On the other hand, lymph-borne VSV captured by SCS macrophages inefficiently replicates in these cells lacking TLR7. Although SCS macrophages can secrete type-I IFN in a TLR7-independent manner, TLR7^−/−^ pDC recruited to SCS layer cannot recognize VSV particles and so fail to produce type-I IFN. Impaired antiviral responses cause VSV dissemination into the central nervous system in the absence of TLR7 function.

Infection of SCS macrophages is critical to protect mice from CNS invasion after peripheral VSV infection (14). In this study, we report for the first time that sensing of VSV via TLR7 receptor is essential for permissiveness of SCS macrophages favoring productive VSV replication and type-I IFN production in draining pLN. This intriguing dual function of TLR7 signaling serves as an important immune check-point in preventing CNS invasion by the virus (Figure 8). Surprisingly, we find that the increased susceptibility of TLR7 deficient mice to VSV neuroinvasion is not due to impaired viral control as VSV infection is reduced in pLN and iLN of TLR7^−/−^ mice after s.c. virus challenge suggesting that TLR7 function is essential for early viral infection in the draining LN. Induction of type-I IFN and pro-inflammatory cytokine responses, on the other hand, are mainly dependent on TLR7 signaling in dLN. In this respect, SCS and medullary macrophages are the central gatekeepers of dLN that encounter microbial breaches of the host skin barrier. SCS macrophages are permissive for VSV replication and induce type-I IFN production, which in turn prevents infection of nerve fibers in the pLN and dissemination of VSV to the CNS (14). Furthermore, clodronate liposome depletion of CD169^+^ macrophages in dLN results in enhanced CD4, CD8 T cell, and neutralizing antibody responses (14). Although humoral and adaptive immune responses are enhanced, mice fail to clear the infection and succumb to lethal ascending paralysis (14). Thus, after lymph borne VSV infection, maintenance of SCS macrophage infection may be a vital step toward curtailing VSV dissemination and protecting mice from neuroinvasion.

In contrast to the dispensable role of humoral and adaptive immunity in conferring full protection, B cells secrete LTα1β2 that maintains the permissiveness of SCS macrophages to VSV replication needed for protection against neuroinvasion (17). Accordingly, we hypothesized that loss of TLR7 signaling specifically in B cells may influence viral loads in the pLN. To this end, our results indicate that TLR7 deficiency in B cells fails to influence VSV infection in the pLN and is dispensable for the reduced virus load observed in the pLN of TLR7^−/−^ mice. This suggests that TLR7-independent pathways may be involved in the induction of LTα1β2 production from B cells. Furthermore, our results show that in addition to TLR7 signaling in hematopoietic cells, TLR7 in non-hematopoietic cells also drives virus replication in the pLN and may provide vital TLR7 survival signals. In line with this finding, previous studies examining the contribution of non-hematopoietic cells in VSV immunity revealed that LYVE^+^ cells in the medulla region of the pLN actively take up VSV after footpad infection (46). Similarly, IFNAR on stromal cells is critical for prevention of ascending hind limb paralysis after lymph borne VSV infection (14), while others have shown that MyD88/Trif/Cardif signaling from stromal cells guarantees full protection after systemic VSV infection (13). In contrast, we find that cell-specific loss of TLR7 in podoplanin^+^ cells fails to influence early viral infection in dLN. The discrepancies in phenotypes between chimeras and conditional TLR7 deficient mice could be explained by incomplete reconstitution of host SCS macrophages with donor BM precursor cells during the reconstitution period (7–8 weeks) adopted in this study. Hashimoto et al. revealed that after lethal irradiation, tissue resident macrophages in the spleen repopulate slowly from donor-derived BM cells, and efficient reconstitution is only achieved ~300 days after engraftment (48). In line with this, SCS macrophages in LN showed more than 80% chimerism after 8–12 weeks of reconstitution (49). Accordingly, our preliminary results demonstrated that approximately 30% of SCS macrophages in pLN of chimeric mice were not replaced with donor-derived precursors in the time frame of reconstitution (data not shown). Hence, remaining host SCS macrophages lacking TLR7 signaling in radio-resistant cells could contribute toward the observed phenotype in chimeras deficient in TLR7 signaling in both compartments. Surprisingly, cell-specific deletion of TLR7 in LysM^+^ cells did not cause a similar reduction in VSV titers in dLN as in TLR7^−/−^ mice although F4/80^+^ peritoneal macrophages are efficiently targeted using LysM-Cre mice (21). In line with these findings, our results showed poor targeting of SCS macrophages in LysM-Cre × RFP mice (data not shown) and no loss of TLR7 gene expression in SCS macrophages in LysM-Cre x TLR7^fl/fl^ mice. This could result as a consequence of low expression of LysM in SCS macrophages. Consistent with our findings, Chtanova et al. determined a restricted co-localization of GFP^+^ and CD169^+^ cells in the SCS layer of uninfected LN using LysM-GFP knock-in mice (47). Moreover, SCS macrophages purified from iLN exhibit low expression of lysosomal enzymes including *Lysozyme M* (49). SCS macrophages and F4/80^+^ medullary macrophages also express CD11c at low levels (49). However, in contrast to impaired VSV titers in TLR7^−/−^ mice, cell-specific deletion of TLR7 function in CD11c^+^ cells resulted in significantly increased viral titers in draining pLN compared to Cre-negative littermates. In line with the findings of Phan et al. (49), our results showed only moderate targeting of *TLR7* gene expression in *ex vivo* sorted SCS macrophages of CD11c-Cre x TLR7^fl/fl^ mice.

VSV inoculated subcutaneously via the footpad drains into the subcapsular LN sinus where CD169^+^ SCS macrophages acquire viral particles (46) and support virus replication (17). In this context, the present findings of our study verify that SCS macrophages in draining pLN retain the highest level of *VSV-N* gene expression among other tested antigen-presenting cells (APC). Furthermore, migratory dDC and to some extend LC show viral gene expression in pLNs of Wt mice. In contrast, in the absence of TLR7 all APC express reduced levels of virus genes. Accordingly, we hypothesized that TLR7 deficiency in skin DC resulted in inefficient transport of the skin-borne VSV particles from the site of infection to dLN. In contrast, our data demonstrate that neither cell-specific deletion of TLR7 in skin-derived Langerin^+^ cells nor complete ablation of Langerin^+^ cells in the foot skin prior to infection reduces the VSV titers in pLN. Furthermore, genetic and pharmacological inhibition of CCR7 and CXCR4 chemokines, respectively, fails to influence lymphoid viral loads. This suggests that subcutaneously inoculated VSV particles are transported to dLN in a TLR7-independent and cell-free manner. In accordance, Manolova et al. demonstrated that large particles (500–2,000 nm) require skin DC to be trafficked to draining LN, whereas small size particles (<200 nm) and virus like particles (30 nm) drain freely to lymphoid tissues (50). Additionally, our results revealed that VSV drains to pLN in a short period of time after s.c. infection as infectious virus was detected in pLN of both Wt and TLR7^−/−^ mice within minutes after infection. In line with this, Junt et al. were able to detect VSV particles in CD169^+^ SCS macrophages in draining pLN within just 5 min of footpad infection using electron microscopy (46). Of note, recent studies reported that trafficking of skin-derived DC to lymphoid organs is a slow process and the numbers of Langerin^+^ dermal DC and LC peaks in draining LN at day 1 and 4 post skin sensitization, respectively (51). Therefore, differences in timing between arrival of VSV particles in pLN shortly after infection and the migration of skin derived DC from the periphery further corroborate our results, suggesting cell-free, and passive transport of skin-borne VSV to pLN.

Since TLR7 deficient mice harbor strongly attenuated VSV loads in the dLN, we queried if virus replication was impaired in LN cells of TLR7^−/−^ mice. To address this point, we first examined VSV replication in *in vitro* generated DC subsets and macrophages. Interestingly, TLR7 deficiency in BM-derived pDC and macrophages resulted in significantly reduced VSV replication but failed to influence virus replication in cDC. Differences in VSV replication in different APC subsets may be due to disparate TLR7 expression in individual APC. Similar to other pro-viral host factors described previously (16, 52), our results demonstrated that the expression of TLR7 was induced in infected SCS macrophages, but not in poorly infected pDC at 12 h p.i. Along these lines, Phan et al. have reported that SCS macrophages express lower levels of surface VSV sensing TLR (TLR4 and TLR13) than medullary macrophages in the naïve state (49). Furthermore, compared to medullary macrophages, SCS macrophages have lower expression of endosomal TLR7, while they express higher levels of cytosolic retinoic acid-inducible gene 1 protein (RIG-I) receptor (49). Furthermore, cytosolic RIG-I receptor was proposed to be the mediator molecule activating IFN production after VSV infection by SCS macrophages (17). Although our results are consistent with previous reports (49) where SCS macrophages of mock-treated mice were found to express low levels of TLR7 receptor, we detected some induction of TLR7 expression in SCS macrophages after VSV infection. Taken together, our data imply that intrinsic TLR7 function in SCS macrophages could contribute to VSV replication in these cells. Indeed, cell-specific deletion of TLR7 function in CD169^+^ cells partially mimicked the attenuated VSV infection in pLN observed in TLR7^−/−^ mice. This partial reduction in viral titers in dLN of CD169-Cre x TLR7^fl/fl^ mice could be explained by moderate targeting efficiency of CD169-Cre in terms of deletion of *TLR7* gene expression in SCS macrophages (Supplementary Figure 9). Iannacone et al. reported that in lymph-borne VSV infection SCS macrophages–and to some extent pDC–are the main immune cells required for neuroprotective type-I IFN production (14). However, our results show that lack of TLR7 function in CD169^+^ cells fails to influence type-I IFN production in pLN after 12 h of s.c. VSV infection. The inconsistency between our results and previous reports may be explained by lower expression of TLR7 as compared to an alternate VSV sensing receptor, RIG-I receptor, in SCS macrophages (49). Hence, while TLR7 is absent in SCS macrophages of CD169-Cre × TLR7^fl/fl^ mice, RIG-I receptor is still functional in these mice, which may trigger the production of type-I IFN at comparable levels to those of Wt littermates. Interestingly, although type-I IFN levels in TLR7^−/−^ and CD11c-Cre x TLR7^fl/fl^ mice were similarly impaired (Supplementary Figure 7A), cell-specific deletion of TLR7 function in CD11c^+^ cells did not result in reduction, but an increase, in viral titers in draining pLN. On the other hand, VSV infection in pLN of CD169-Cre x TLR7^fl/fl^ mice displayed a pattern similar to that observed in TLR7^−/−^ mice although type-I IFN production was still intact in the former. In line with these findings, Iannacone et al. showed that depletion of SCS macrophages caused a significant reduction in both VSV load and IFN levels in dLN indicating that absence of type-I IFN does not increase the infection in other lymphoid cells, but it is crucial to control peripheral nerve infection (14). Collectively, our results suggest that TLR7, but not type-I IFN, plays a role in controlling the permissiveness of SCS macrophages for early VSV replication in dLN.

In conclusion, we have identified a previously unknown function of TLR7 in ssRNA virus replication within productively infected SCS macrophages using a lymph-borne VSV infection model. Our findings not only expand the knowledge about the mechanisms by which TLR7 controls the onset of VSV-induced CNS disease but also indicate the existence of other non-dispensable roles aside of cytokine induction and PRR function in viral infections. Finally, our identification of TLR7 as a host factor sustaining viral replication may serve as a potential target combined with IFN boosting adjuvants to prevent the establishment of early ssRNA pathogenic viral infections like rabies virus in humans.

## Data Availability

All datasets generated for this study are included in the manuscript and/or the supplementary files.

## Ethics Statement

All animal experimental procedures were performed in compliance with the German animal protection law (TierSchG BGBl. I S. 1105; 25.05.1998) and were approved by the Lower Saxony Committee on the Ethics of Animal Experiments and the responsible state office (Lower Saxony State Office of Consumer Protection and Food Safety) under permit numbers 33.9-42502-04-12/1020 and 33.9-42502-04-09/1785.

## Author Contributions

GS and FP designed and conducted the experiments and wrote the manuscript. MF, ML, MG, and MS conducted the experiments. UK provided the VSV strains used in this study. VD, VK, and KL performed the histology and immunofluorescence imaging. BL provided the Podoplanin-Cre (23). BC generated and provided the Langerin-Cre (24) and Langerin-DTR:eGFP (25) mice. HW and TS in collaboration with Taconic Artemis generated the TLR7^fl/fl^ mice. GS and MF analyzed the data and prepared the figures. FP and TS supervised the work. TS conceived the study and acquired funding. All authors edited the manuscript.

### Conflict of Interest Statement

The authors declare that the research was conducted in the absence of any commercial or financial relationships that could be construed as a potential conflict of interest.
