# Supplementary material for: TLR7 Controls VSV Replication in CD169+ SCS Macrophages and Associated Viral Neuroinvasion
Source: Front Immunol. 2019 Mar 15;10:466. doi: 10.3389/fimmu.2019.00466 (PMC6428728; doi:10.3389/fimmu.2019.00466)
Supplement: Supplementary file 1 [file Data_Sheet_1.pdf]

Supplementary Material

TLR7 Controls VSV Replication in CD169$^+$ SCS Macrophages and Associated Viral Neuroinvasion

Gülhas Solmaz[1#], Franz Puttur[1#,*], Marcela Francozo[1], Marc Lindenberg[1], Melanie Guderian[1], Maxine Swallow[1], Vikas Duhan[2], Vishal Khairnar[2], Ulrich Kalinke[3], Burkhard Ludewig[4], Björn E. Clausen[5], Hermann Wagner[6], Karl S. Lang[2] and Tim D. Sparwasser[1,7*]

*** Correspondence:**
Prof. Dr. Tim D. Sparwasser
Email: sparwasser.office@uni-mainz.de

Dr. Franz Puttur
Email: f.puttur@imperial.ac.uk

SUPPLEMENTARY FIGURE 1

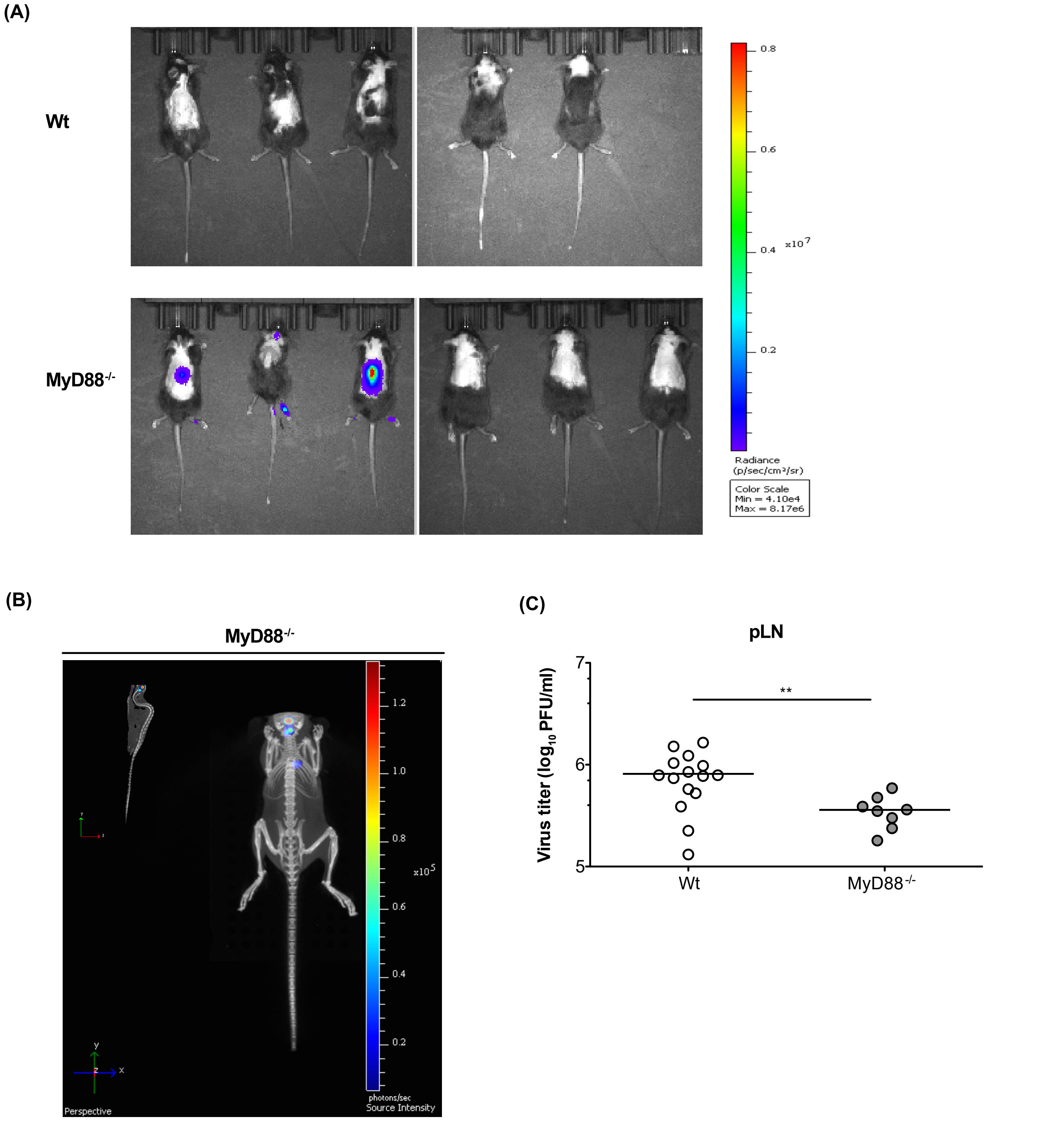

Supplementary Figure 1. MyD88 protects mice from peripheral VSV infection induced fatal CNS invasion. **(A-B)** VSV dissemination into the CNS of MyD88$^{-/-}$ mice displayed as **(A)** viral bioluminescence alone or **(B)** an overlay with mouse computer tomography (CT) at day 10 post VSV-luciferase infection (n=5-6 mice/group). Results are representative data from **(B)** two or **(A)** three independent experiments showing similar results. **(C)** VSV titers in pLN of Wt and MyD88$^{-/-}$ mice at 12h post s.c. VSV-Indiana (5 x 10^5 pfu) infection. Results are pooled from two independent experiments (n=4-8 mice /group /experiment). The significance of differences between groups was analyzed by two-tailed t-test. ** $p< 0.01$.

SUPPLEMENTARY FIGURE 2

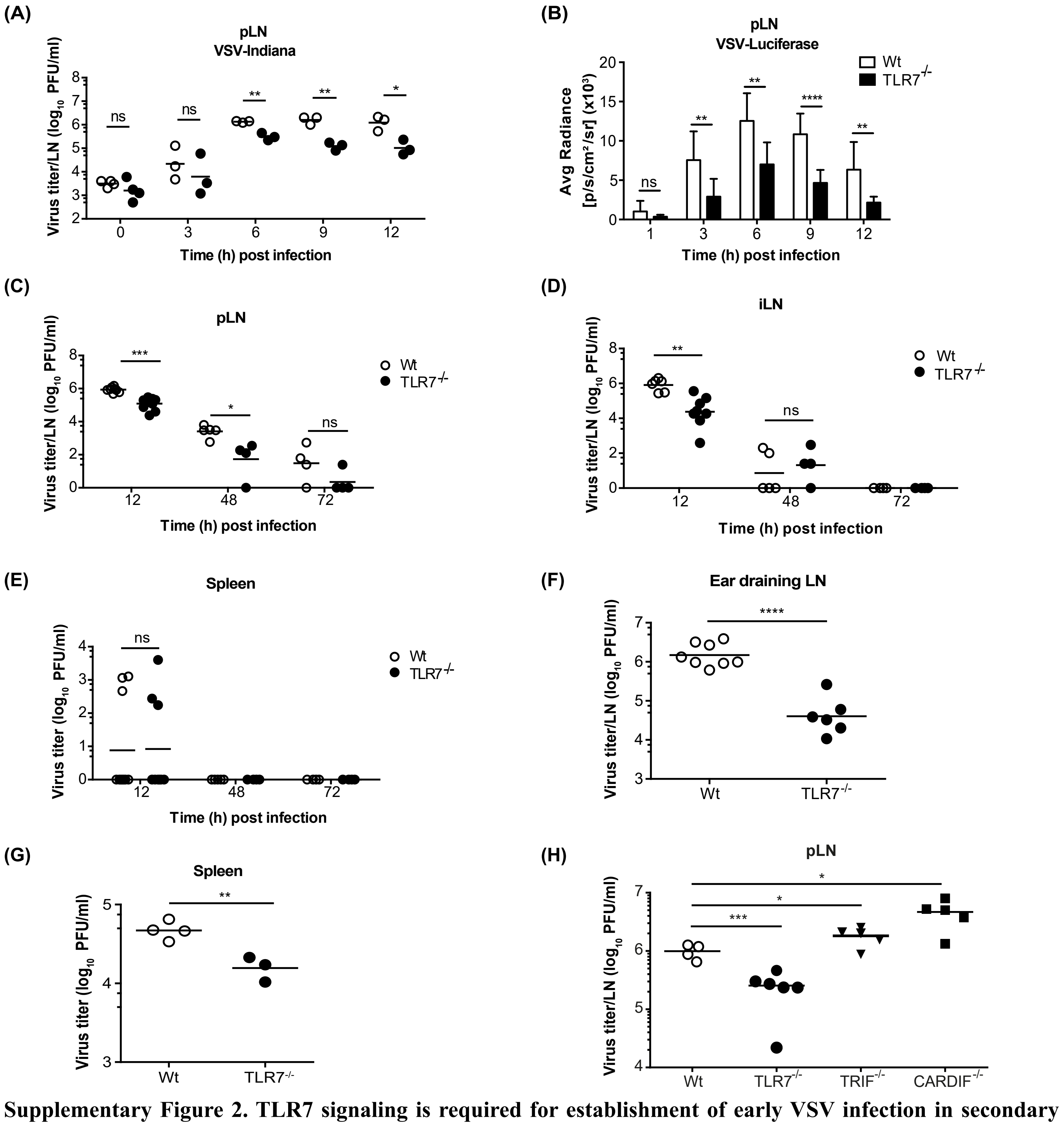

Supplementary Figure 2. TLR7 signaling is required for establishment of early VSV infection in secondary lymphoid organs, but dispensable for clearance of VSV at later time points of infection. **(A-B)** Early time kinetics of **(A)** VSV-Indiana (n= 3-4 mice/group/experiment) and **(B)** VSV-Luciferase (n= 3-6 mice/group/experiment) infection in pLN of Wt and $TLR7^{-/-}$ mice assessed at corresponding time points by standard plaque assay or in vivo imaging system, respectively. Data are from one of two individual experiments with similar results. **(C-E)** Kinetics of VSV titers in **(C)** pLN, **(D)** iLN and **(E)** spleen of control and $TLR7^{-/-}$ mice infected with VSV-Indiana (5 x 10^5 pfu) subcutaneously via the foot skin. Data are obtained from one individual experiment and results were shown as mean of 4-8 mice per group. **(F)** Viral loads in ear draining LNs of Wt and $TLR7^{-/-}$ mice infected with VSV-Indiana intradermally via the ear pinna. VSV titers were assessed at 12 h p.i. Results are depicted as mean values and pooled from two independent experiments where 2-4 mice per group were used. **(G)** Viral titers in the spleen of mice 12 h after intravenous VSV-Indiana infection via the tail vein (N=1). **(H)** Viral titers in pLN of mice at 12h post VSV-Indiana infection. Results were depicted as mean. (N=1. n=4-7 mice/group). Significance of differences between groups was analyzed by two-tailed t test (A,C-H) or Mann-Whitney test (B). ns: non-significant, *: $p < 0.05$, **: $p < 0.01$, *** $p < 0.001$, ****: $p < 0.0001$.

SUPPLEMENTARY FIGURE 3

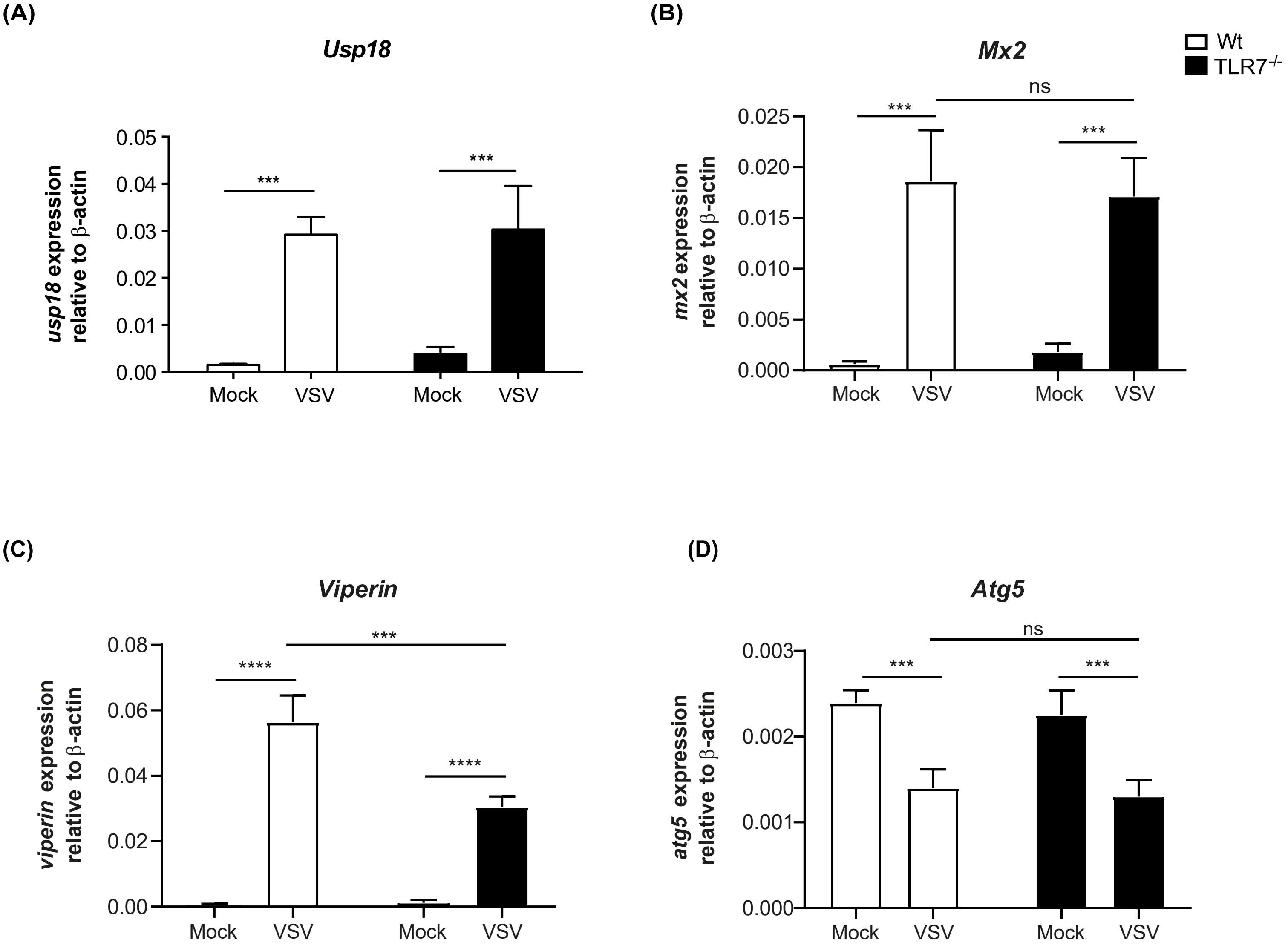

Supplementary Figure 3. Expression of genes known to regulate type-I IFN pathway and virus replication. (A-D) Expression of **(A)** *Usp18,* **(B)** *Mx2,* **(C)** *Viperin and* **(D)** *Atg5* mRNAs in pLNs of Wt and TLR7$^{-/-}$ mice at 12h post-PBS treatment or VSV-Indiana infection. Results were assessed relative to housekeeping β-actin gene and depicted as mean ± standard deviation. (n=3-5 mice/group/experiment). Data are from one of **(B-D)** two or **(A)** three individual experiments showing similar results. Significance of differences between groups was analyzed by Two-Way ANOVA. ns: non-significant, *** p< 0.001, ****: p < 0.0001.

SUPPLEMENTARY FIGURE 4

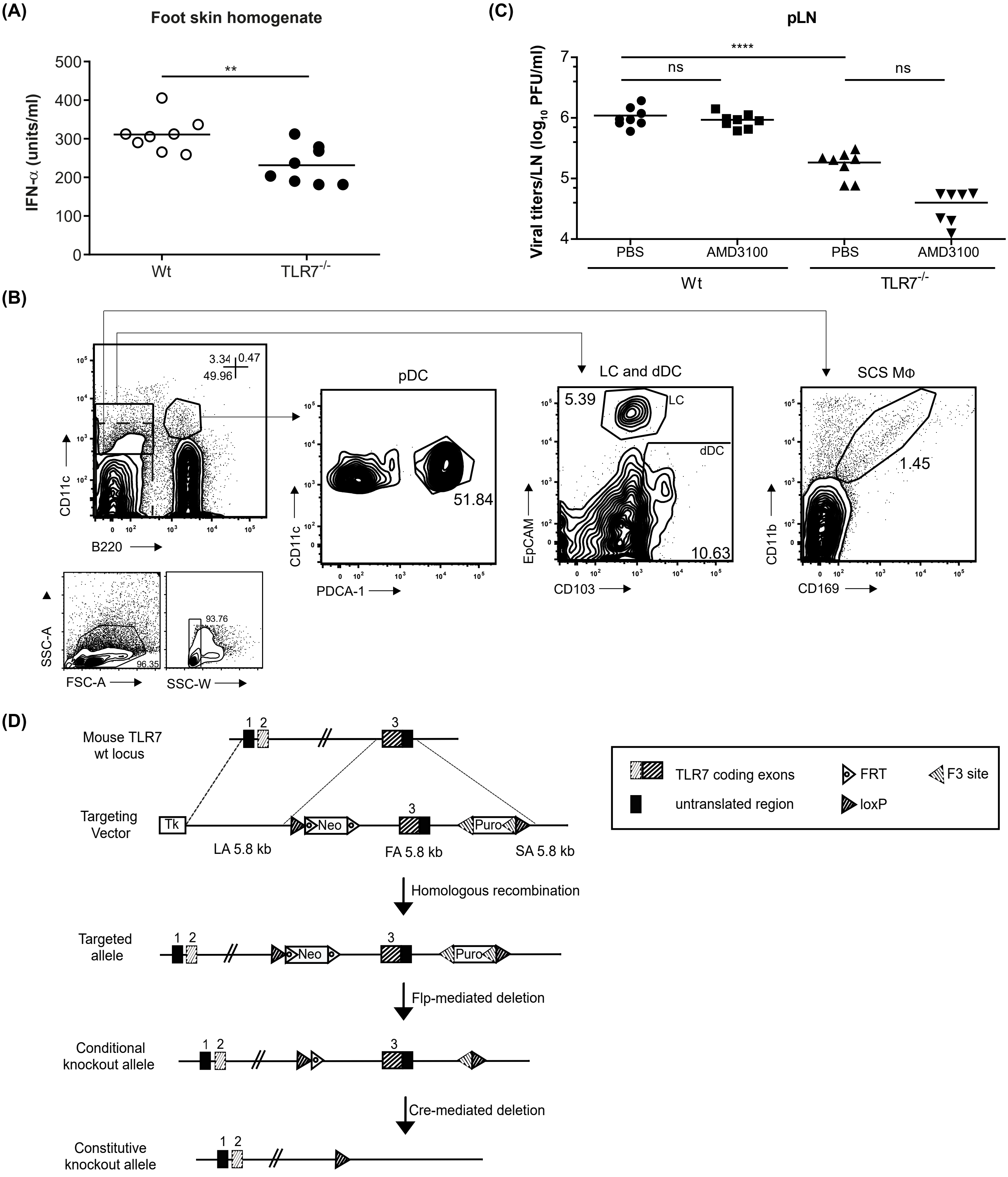

Supplementary Figure 4. Novel transgenic TLR7$^{fl/fl}$ mouse model.
(A) IFN-α concentrations in foot skin homogenate of mice infected subcutaneously with VSV-Indiana s.c. at 12h p.i. **(B)** Sorting strategy for SCS MΦ (CD11c$^{-/int}$ B220^{-} CD11b^{+} CD169^{+}), pDC (CD11c^{int} B220^{+} PDCA-1^{+}), skin derived LC (CD11c^{int} B220^{-} EpCAM^{+} CD103^{-}) and CD103^{+} dDC (CD11c^{int} B220^{-} EpCAM^{-} CD103^{+}) populations from Wt and TLR7$^{-/-}$ mice at 12 h post s.c. PBS treatment or VSV-Indiana (5 x 10^5 pfu) infection. **(C)** VSV titers in pLN of Wt and TLR7$^{-/-}$ mice treated subcutaneously with 100 μg of AMD3100 one hour before Wt VSV infection (5 x 10^5 pfu) and assessed at 12 hr p.i. Data are pooled from two individual experiments with similar results (n= 4 mice/group/ experiment). **(D)** Schematic diagram showing construction of a conditional allele of *TLR7* gene flanking exon 3 with loxP sites. Significance of differences between groups was analyzed by One-Way ANOVA. ns: non-significant, **: $p < 0.01$, ****: $p < 0.0001$.

SUPPLEMENTARY FIGURE 5

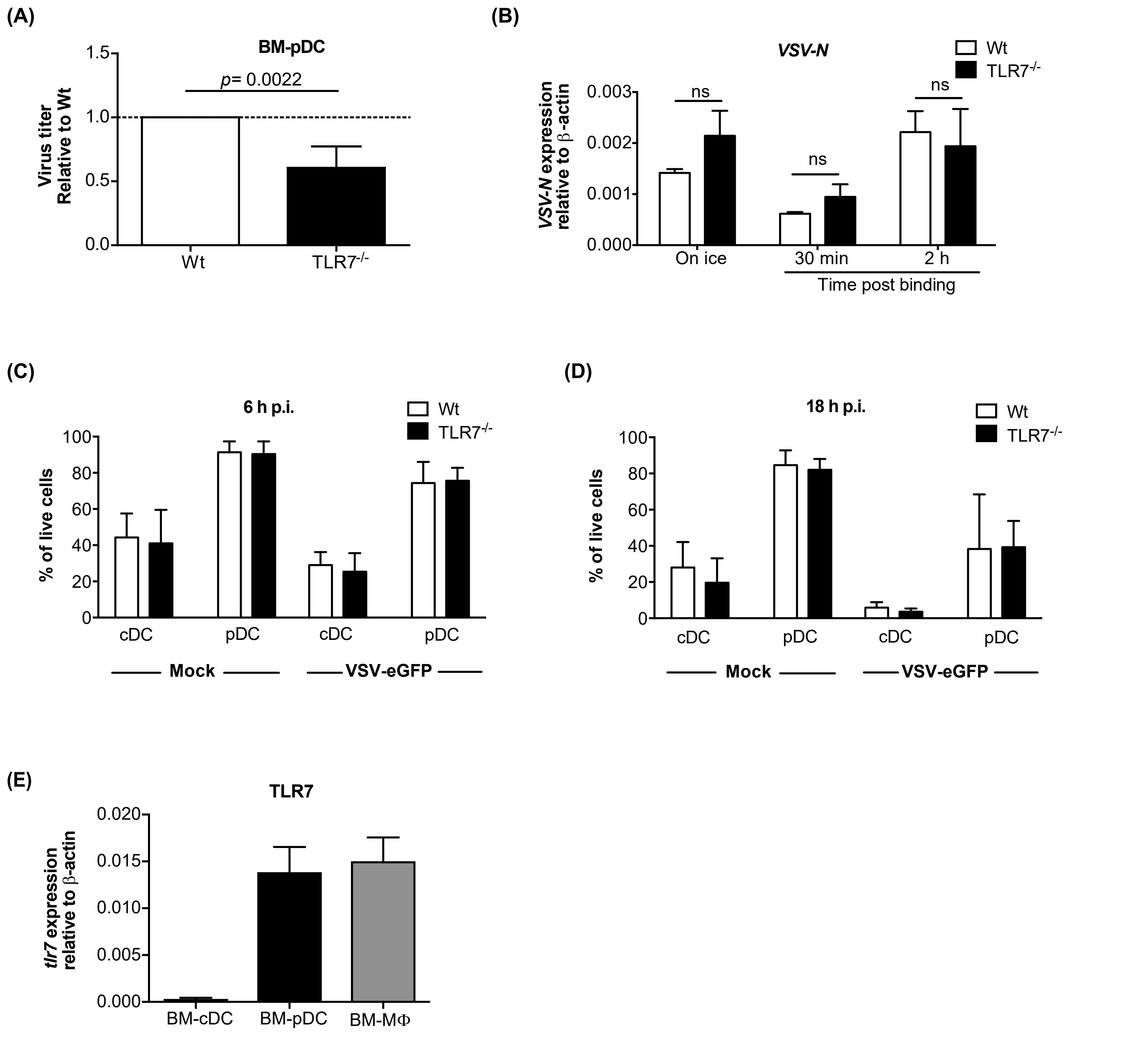

Supplementary Figure 5. TLR7 signaling is redundant for binding and uptake of VSV in bone marrow-derived pDC in vitro.

(A-B) Flt3-L generated and FACS-sorted Wt and TLR7$^{-/-}$ BM-pDC infected with VSV-Indiana (MOI 5). **(A)** VSV titers within BM-pDC at 6hr p.i. quantified by standard plaque assay following cell lysis with handheld homogenizer. Data are normalized to VSV titer levels in Wt pDC and pooled from six individual experiments with similar results. **(B)** Expression of *VSV-N* mRNA in BM-pDC. Directly after addition of viral inoculum onto the cells, cells were kept on ice for one hour and washed with PBS. Virus binding on the cell was determined using the pDC kept on ice. For uptake assay, washed pDC were fed with fresh medium and incubated for 30 min or 2h. Cells were washed, lyzed and the amount of VSV taken up by Wt and TLR7$^{-/-}$ pDC were assessed by qPCR at corresponding time points. Data is representative of two individual experiments with similar results. Results show mean ± standard deviation values. **(C-D)** The viability of in vitro generated Flt3-L driven cDC and pDC upon PBS (Mock) treatment or VSV-eGFP infection (MOI 10) at **(C)** 6h and **(D)** 18h p.i. determined by flow cytometry. Results are pooled from three independent experiments. **(E)** Expression of *TLR7* mRNA in Flt3-L generated and FACS-sorted BM-derived Wt cDC, pDC and LCCM generated BM-derived macrophages at naïve state. Results were assessed relative to expression of the housekeeping gene β-actin and depicted as mean ± standard deviation. Data is pooled from two individual experiments with similar results. Significance of differences between groups was analyzed by (A) one-sample t-test or (B) Two-Way ANOVA.

SUPPLEMENTARY FIGURE 6

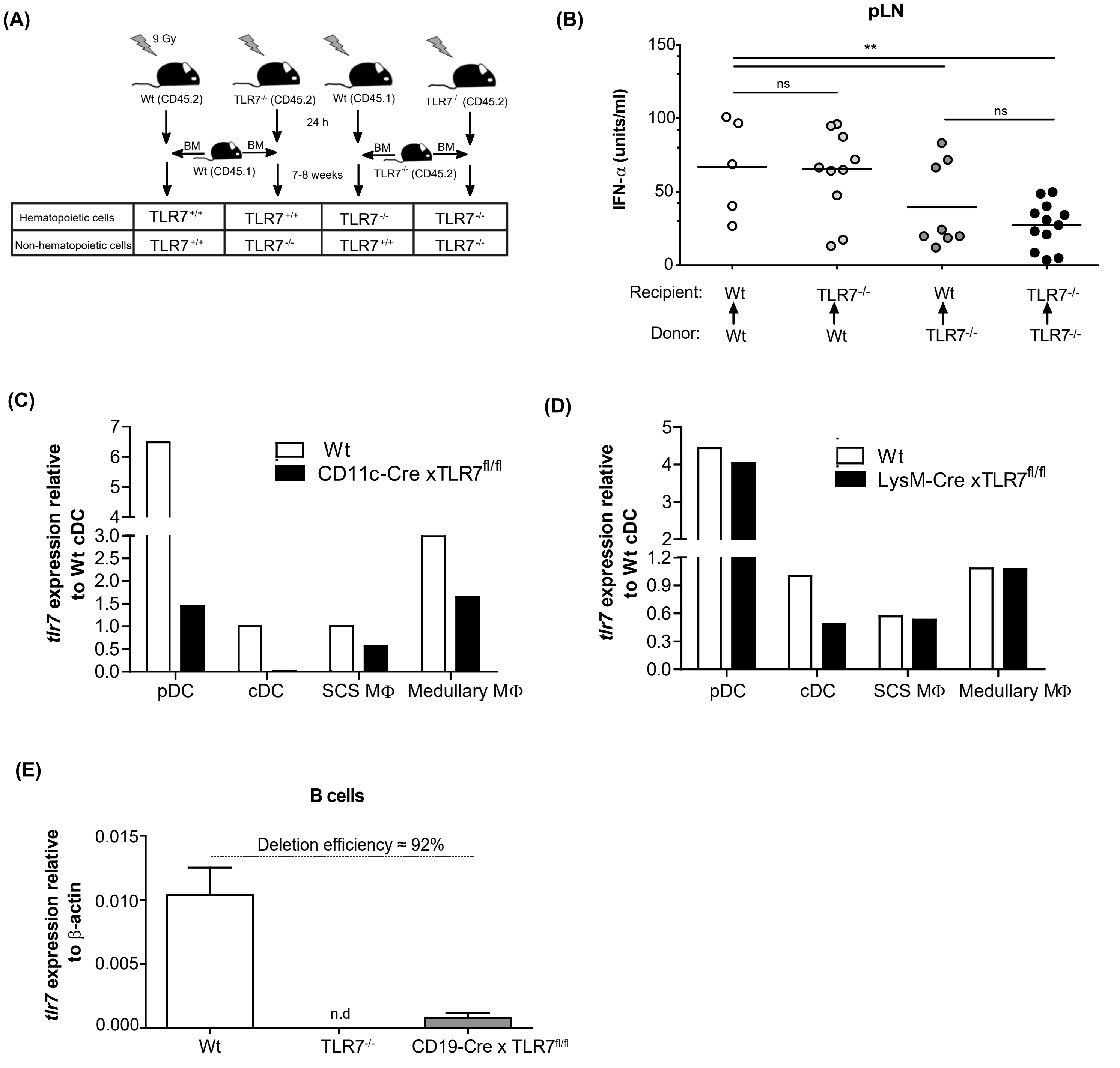

Supplementary Figure 6. Cell specific deletion of TLR7 function by using the novel transgenic TLR7$^{fl/fl}$ mouse model.

(A) Schematic illustration of experimental setup for generation of BM chimeric mice. **(B)** IFN-α concentrations in pLN homogenate of BM chimeric mice infected subcutaneously with VSV-Indiana s.c. at 12h p.i. Results are pooled from two independent experiments. Results were depicted as mean. **(C-D)** Expression of *TLR7* mRNA in ex vivo isolated and FACS-sorted dLN-resident pDC, cDC, SCS macrophages and medullary macrophages of **(C)** CD11c-Cre x TLR7$^{fl/fl}$ and **(D)** LysM-Cre x TLR7$^{fl/fl}$ mice. **(E)** Deletion efficiency of *TLR7* gene in B cells MACS-sorted from iLN of Wt, TLR7$^{-/-}$ and CD19-Cre x TLR7$^{fl/fl}$ mice was assessed by qPCR. Data are from one experiment having 2-3 biological replicates. Results show mean ± standard deviation. n.d: not-detected. Significance of differences between groups was analyzed by two-tailed t test. ns: non-significant, **: $p < 0.01$.

SUPPLEMENTARY FIGURE 7

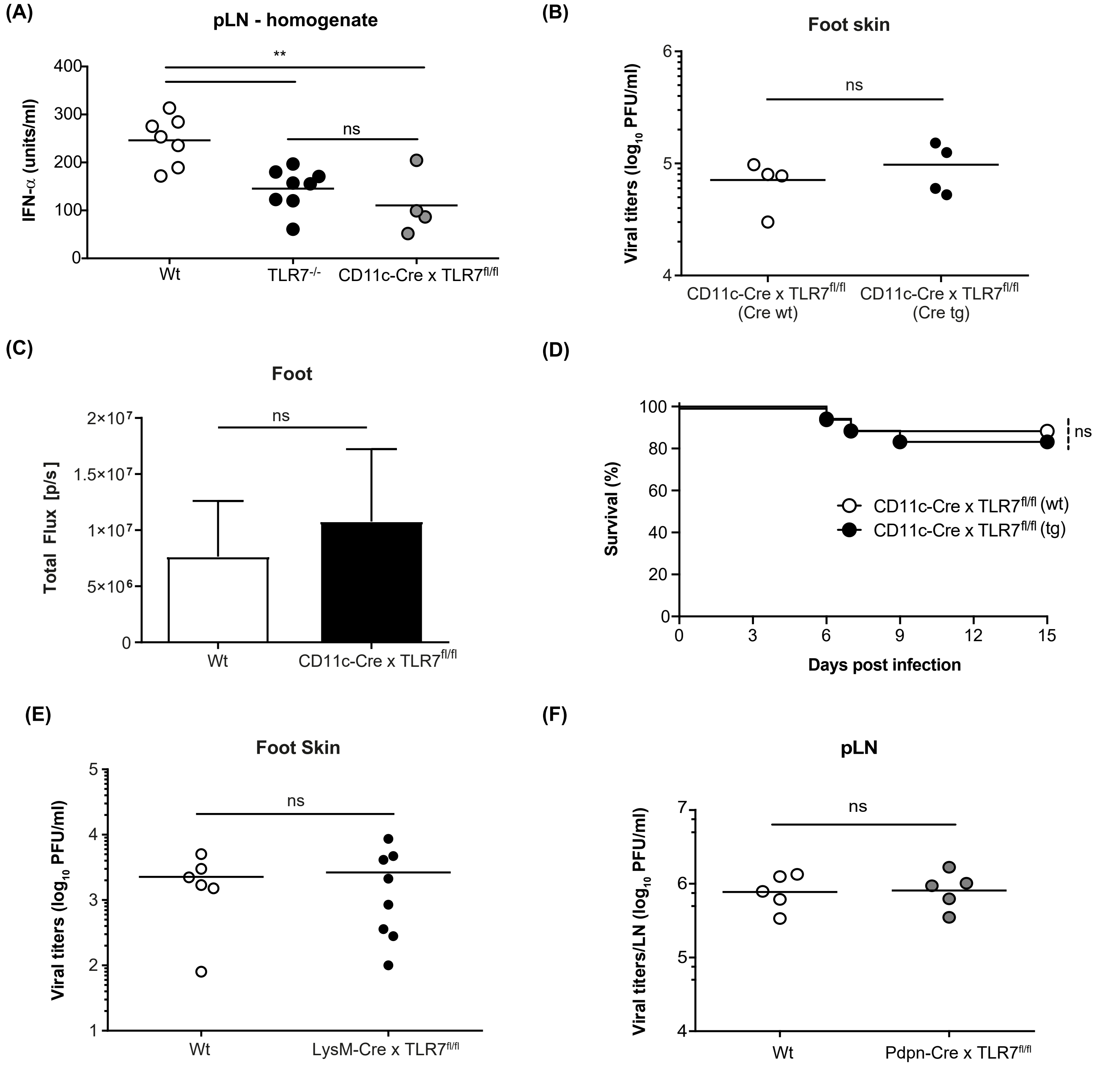

Supplementary Figure 7. TLR7 function in individual cell populations to control virus infection
(A) IFN-α concentrations in pLN homogenate of Wt, TLR7$^{-/-}$ and CD11c-Cre x TLR7$^{fl/fl}$ mice at 12h post s.c. VSV-Indiana infection (N=1, n=4-8 mice/group). **(B)** Virus load in foot skin of control (Cre wt) and CD11c-Cre x TLR7$^{fl/fl}$ (Cre tg) mice infected s.c. with VSV-Indiana at 12h p.i. (N=1). **(C)** Total flux measured on feet of mice 12h after s.c. 5 x 10^5 pfu of VSV-luciferase infection assessed by IVIS (N=1. n=6-7 mice/group) **(D)** Survival curves of Cre negative (wt, n=17) and positive (tg, n=19) CD11c-Cre x TLR7$^{fl/fl}$ mice infected s.c. with 5 x 10^6 pfu of VSV-Indiana. Shown here are pooled data of two independent experiments with similar results. **(E)** Virus load in foot skin of Wt and LysM-Cre x TLR7$^{fl/fl}$ mice infected s.c. with VSV-Indiana at 12h p.i. (N=1). **(F)** VSV titers in pLN of Pdpn-Cre x TLR7$^{fl/fl}$ mice and cre negative littermates (Wt) at 12h p.i. Data are pooled from two individual experiments with similar results. Significance of differences between groups was analyzed by (A-C, E-F) two-tailed t test or (D) Log-rank test. ns: non-significant, **: $p < 0.01$.

SUPPLEMENTARY FIGURE 8

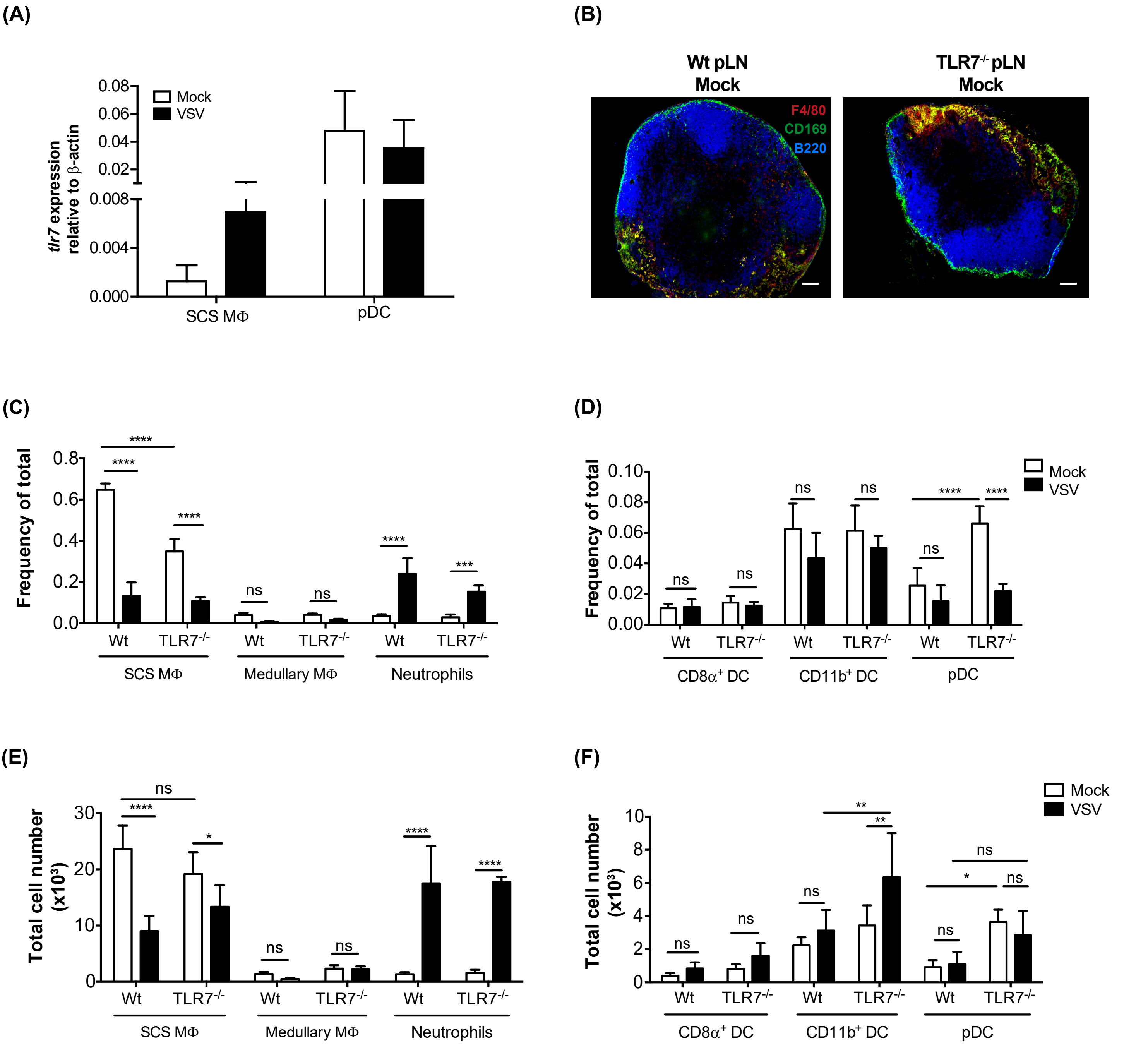

Supplementary Figure 8. Absence of TLR7 does not cause remarkable modifications in leukocyte profile and architecture of draining pLN.
(A) Expression of *TLR7* mRNA in ex vivo isolated and FACS-sorted pLN resident SCS macrophages and pDC of Wt mice either treated with sterile PBS (Mock) or infected with VSV-Indiana subcutaneously. Results were assessed relative to expression of the housekeeping gene β-actin and depicted as mean ± standard deviation. Data are pooled from four individual experiments with similar results. **(B)** Representative fluorescent microscopy images (10x) of pLN of Wt and TLR7$^{-/-}$ mice upon s.c. sterile PBS (Mock) treatment via the foot skin. Scale bars reflect 100µm. Results are representative of two independent experiments (n=1-4 mice/group/experiment). **(C-D)** Frequency of total **(C)** macrophage (MΦ) subsets and neutrophils, and **(D)** DC subsets within the lymphocytes. **(E-F)** Total cell numbers of **(E)** macrophage (MΦ) subsets and neutrophils, and **(F)** DC subsets in pLNs of mice 12h after s.c. mock treatment or VSV-Indiana infection. Results show mean ± standard deviation. Data are from one of two independent experiments with similar results. Significance of differences between groups was analyzed by Two-Way ANOVA with Bonferroni post-test. ns: non-significant, *: $p < 0.05$, **: $p < 0.01$, ***: $p < 0.001$, ****: $p < 0.0001$.

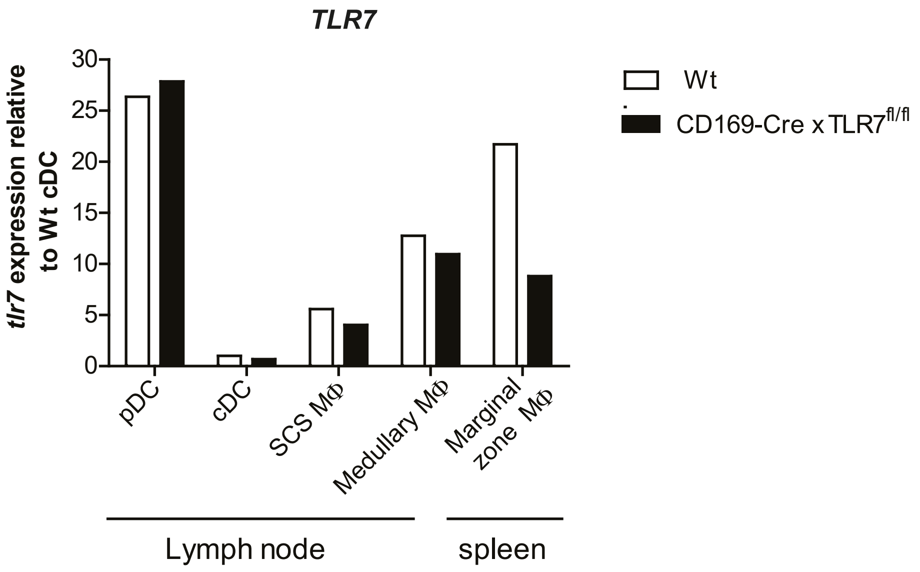

Supplementary Figure 9. TLR7 gene expression in SCS macrophages of CD169-Cre x TLR7$^{fl/fl}$ mice is moderately targeted.

TLR7 mRNA expression levels in ex vivo isolated and FACS-sorted dLN resident pDC, cDC, SCS macrophages and medullary macrophages, and splenic marginal zone macrophages of Wt and CD169-Cre x TLR7$^{fl/fl}$ naïve mice. Data are from one of two independent experiments with similar results.
